# Adaptive immunity selects against malaria infection blocking mutations

**DOI:** 10.1371/journal.pcbi.1008181

**Published:** 2020-10-08

**Authors:** Bridget S. Penman, Sylvain Gandon

**Affiliations:** 1 Zeeman Institute and School of Life Sciences, University of Warwick, Coventry, United Kingdom; 2 CEFE, CNRS, University of Montpellier, Paul Valéry University of Montpellier, EPHE, IRD, Montpellier, France; University of Chicago, UNITED STATES

## Abstract

The mutation responsible for Duffy negativity, which impedes *Plasmodium vivax* infection, has reached high frequencies in certain human populations. Conversely, mutations capable of blocking the more lethal *P*. *falciparum* have not succeeded in malarious zones. Here we present an evolutionary-epidemiological model of malaria which demonstrates that if adaptive immunity against the most virulent effects of malaria is gained rapidly by the host, mutations which prevent infection *per se* are unlikely to succeed. Our results (i) explain the rarity of strain-transcending *P*. *falciparum* infection blocking adaptations in humans; (ii) make the surprising prediction that mutations which block *P*. *falciparum* infection are most likely to be found in populations experiencing low or infrequent malaria transmission, and (iii) predict that immunity against some of the virulent effects of *P*. *vivax* malaria may be built up over the course of many infections.

## Author summary

Malaria has profoundly influenced human evolution. However, humans have adapted to two major malaria parasites, *Plasmodium falciparum* and *Plasmodium vivax*, by strikingly different strategies. The Duffy negative blood group impairs *P*. *vivax* entry to red blood cells and reduces the chance of becoming infected. No equivalent blocking adaption against *P*. *falciparum* has evolved to become widespread, despite the existence of candidate mutations. We show that rapidly gained adaptive immunity to malaria virulence limits the success of infection blocking mutations. This could account for humans’ contrasting adaptations to *P*. *falciparum* and *P*. *vivax*. Our results highlight the critical role of adaptive immunity in determining how vertebrate hosts evolve in response to pathogen selection, and provide a new framework within which to understand this process.

## Introduction

Rapidly evolving parasites exert strong selective pressures on their hosts. Malaria parasites (red blood cell infecting apicomplexan parasites of the genus *Plasmodium*) have had a profound effect on human genetics [1] (Table 1). Of human-infecting malaria parasites, *Plasmodium falciparum* and *Plasmodium vivax* present the greatest public health concern. *P*. *falciparum* is more strongly associated with coma[2] and death [3,4] than *P*. *vivax*, and historically *P*. *vivax* was believed to be relatively benign [5]. Nevertheless, *P*. *vivax* is now recognised as a potentially lethal infection [5–9].

**Table 1:** Known human adaptations to malaria.

Protein	Locus	Evidence for malaria selection	Malaria infection impeding potential	Notes on infection blocking properties
Haemoglobin	*HBA* and *HBB*	Mutations in *HBB* and *HBA* including sickle cell, alpha thalassaemia and haemoglobin C protect against severe *P*. *falciparum* disease [55]. Mutations in *HBB* and *HBA* reach elevated frequencies in old world malarious regions [56].	Low	Gong *et al* found that sickle cell trait offers no inherent protection against acquiring blood stage *P*. *falciparum* infection *per se* without concurrent adaptive immunity [57]. Mangano *et al* demonstrated that sickle cell heterozygosity and haemoglobin C homozygosity offer some protection against *P*. *falciparum* parasitaemia [58], which did not appear to be entirely due to adaptive immunity, but the effect they observed could be caused by enhanced clearance of parasitaemia rather than reduced probability of initial infection. Rosanas-Urgell *et al* observed no difference in *P*. *falciparum* infection rates associated with alpha thalassaemia in children aged 3–21 months [59]. Lin *et al* observed a protective effect of alpha thalassaemia against *P*. *falciparum* infection in children aged 5–14 years [60], but taken together with Rosasnas-Urgell’s result this could also be an example of a protective effect due to adaptive immunity rather than an inherent property of alpha thalassaemia. Alpha thalassaemia may even increase the probability of blood stage malaria infection in young children, for both *P*. *vivax* and *P*. *falciparum*, with the effect more pronounced for *P*. *vivax* [61]. This may be because alpha thalassaemia is associated with reticulocytosis. *P*. *vivax* only infects reticulocytes, and *P*. *falciparum* prefers to infect younger red blood cells. *In vitro* studies show only small reductions in *P*. *falciparum* invasion for sickle cell heterozygous and haemoglobin C homozygous red blood cells [62,63], and no effect of alpha thalassaemia on *P*. *falciparum* red blood cell invasion [64]. However, heterozygosity (but not homozygosity) for the HBB mutation haemoglobin E is associated with a much smaller pool of *P*. *falciparum* “invadable” red blood cells than normal *in vitro* [65], as measured by an invasion selectivity index.
Glucose-6-phosphate dehydrogenase	*G6PD*	Point mutations in the *G6PD* gene on the X chromosome result in lower activity forms of the glucose-6-phosphate dehydrogenase (G6PD) enzyme. The haplotypic diversity of these mutations suggests they have a recent origin in human populations, in keeping with malaria selection [66], and there is an average frequency of 8% G6PD deficiency across malaria endemic countries [67]. Some studies have suggested that hemizygous males [68] and perhaps both female heterozyogtes and male hemizygotes for a G6PD deficiency mutation are protected against severe *falciparum* malaria [69]. However, a recent meta-analysis of the protective effect of G6PD deficiency found only a protective effect of heterozygosity against uncomplicated *falciparum* malaria, limited to African populations [70].	Low	*In vitro* experiments using four different *P*. *falciparum* strains found no differences in erythrocyte invasion between normal and glucose-6-phosphate dehydrogenase deficient blood [71].
Complement receptor 1	*CR1*	Complement receptor 1 is used by *P*. *falciparum* to enter red blood cells. It has recently been shown that the Sl2 exon 29 variant of *CR1* (which is frequent in sub Saharan Africa) protects against uncomplicated and cerebral malaria, but only in the absence of alpha thalassaemia [72]	Low (but not all polymorphisms investigated)	The nucleotide 3650 G/A SNP in exon 22 of *CR1*, which has been associated with lower CR1 expression, does not affect the probability of PCR-detectable *P*. *falciparum* infection[60].
Band 3	*SLC4A1*	Southeast Asian ovalocytosis (SAO), also known as Melanasian ovalocytosis, results from heterozygosity for a deletion of 27 nucleotides from *SLC4A1* (and hence a deletion of 9 amino acids in band 3 protein) [73]. This deletion is lethal in the homozygous state. Heterozygotes seem to be protected against cerebral malaria caused by *P*. *falciparum* [74] and the distribution of the deletion in Papua New Guinea is broadly consistent with malaria selection [75].	Strain specific	*In vitro* studies suggest SAO may impede the invasion of certain *P*. *falciparum* strains, possibly those requiring an as yet un identified chymotrypsin sensitive receptor [76], but the same study shows SAO does not block red blood cell invasion by all *P*. *falciparum* strains.
Glycophorins A, B and C	*GYPA*, *GYPB*, *GYPC*	The Dantu blood group is caused by a duplicated hybrid GYPA/GYPB gene, is associated with a reduction in the risk of severe *P*. *falciparum* malaria, and reaches frequencies of >10% in Kenya [77]. GYPB polymorphism is associated with the risk of *P*. *falciparum* infection in the Brazilian Amazon [78]. A GYPC exon 3 deletion known to reduce invasion by *P*. *falciparum* parasites which use EBA140 to enter red blood cells is observed at a high frequency in coastal Papua New Guinea [79].	Partial (Dantu blood group)	The glycophorin invasion pathway is not essential for *P*. *falciparum* blood stage infection: certain strains can readily adapt to employ non glycophorin-dependent pathways [80,81]. Spontaneous switching between using glycophorins for red blood cell entry and not using glycophorins for entry has recently been observed in *P*. *falciparum* grown in suspended rather than static cultures [82]. *In vitro* experiments show that Dantu red blood cells are invaded to a lower extent by all strains of *P*. *falciparum* tested, regardless of their reliance on binding glycophorins for cell entry [83]. Higher membrane tension was shown to reduce invasion in non-Dantu red blood cells as well as Dantu cells, and Dantu red blood cells had a higher membrane tension on average [83].
Duffy antigen	*FY* (*DARC*)	Sub Saharan African populations display extremely high frequencies of a mutation (FY*O) which eliminates erythrocytic expression of the Duffy antigen [84]. The population genetics of variation at the *FY* locus is consistent with a FY*O mutation sweeping to fixation in sub Saharan African populations within the last 42 thousand years, from a standing variation frequency of 0.1% [20].	High	Human challenge studies during the mid 20^th^ century showed that Duffy negative individuals (homozygous for FY*O) are highly resistant to infection with *P*. *vivax* [11]. *Vivax* infection in Duffy negative individuals has subsequently been detected [13–16]. However, infection occurs at a lower rate than in Duffy positive individuals in the same populations. Duffy negativity offers 49–69% protection when infection is measured by PCR positivity in an asymptomatic population, or 96–98% protection when infection is measured by PCR positivity in symptomatic patients in a clinical setting [16]. Other studies have shown that the A variant of the Duffy antigen (Fy^a^) is associated with weaker binding of Duffy Binding Protein than the B variant of the Duffy antigen (Fy^b^)[85], and a lower probability of experiencing clinical infection [86]. Heterozygosity for FY*O and FY*A is associated with a reduced probability of PCR detectable *vivax* infection[24] but it is less clear that heterozygosity for FY*O and FY*B is associated with a reduced probability of *vivax* infection [85,86].

Known human adaptations to *P*. *falciparum* or *P*. *vivax* are summarised in Table 1. Multiple adaptations to *P*. *falciparum* exist, but few affect the probability of becoming infected *per se*. Instead, *P*. *falciparum* adaptations mainly reduce the probability of dying from the infections that occur. By contrast, Duffy negativity, an adaptation to *P*. *vivax*, substantially impairs infection [10–16]. The lack of an infection blocking adaptation to *P*. *falciparum* is not simply due to an absence of candidate human loci. Basigin, Complement Decay Accelerating Factor (CD55) and the Langereis blood group antigen (ABCB6) are red blood cell invasion receptors required by all *P*. *falciparum* strains [17–19]. Red blood cells with naturally occurring genetic variants of these loci (the OK^-^ blood group; the Inab phenotype and Lan null red blood cells) are resistant to *P*. *falciparum* invasion *in vitro* [17–19]. However, there is no evidence that mutations at any of these loci have reached high frequencies in any population under strong malaria selection.

Selection, presumably involving *P*. *vivax*, elevated the frequency of the mutation responsible for Duffy negativity (FY*O) to near fixation throughout much of sub Saharan Africa within the last 49000 years [20]. Mutations in basigin, CD55 or ABCB6 could block the more lethal *P*. *falciparum*, but this evolutionary opportunity does not appear to have been exploited in human populations. Why should this be the case? We present and analyse a new evolutionary-epidemiological model of malaria to explore this question.

### Model summary

**Fig 1:**
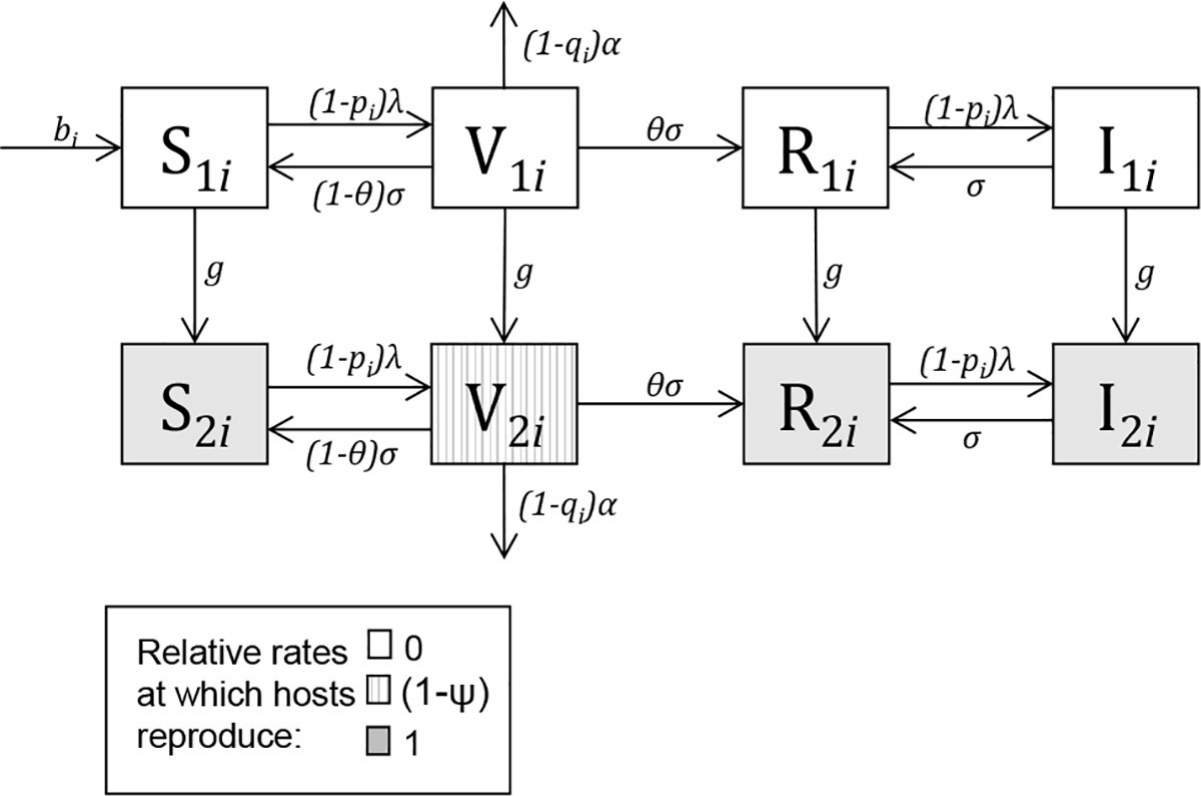
The compartmental model. Hosts of genotype *i* are compartmentalised into immature and mature susceptible hosts (S_1_ and S_2_); immature and mature virulently infected hosts (V_1_ and V_2_); immature and mature hosts resistant to virulence (R_1_ and R_2_), and finally immature and mature hosts who are infectious but not at risk of virulence (I_1_ and I_2_). The compartmental model is fully described by Eqs 1–8 in the Methods. A host transitions between compartments at the rates indicated on each arrow. The force of infection (*λ*) is given in Eq 9, and the birth rate (*b*_*i*_) is given by Eq 10. All parameters of the model, which include the other rates in this diagram, are defined in Table 2. All hosts die from a background death rate (*μ*) which has not been visualised here.

The epidemiological process in our model takes the form S-V-R-I-R. Fig 1 illustrates the compartmental structure of the model. Equations describing the model, and a full description of model parameters, are given in the Methods. The parameters of the model are summarised in Table 2. Hosts are born susceptible (state S). When susceptible hosts become infected they suffer virulent infections (state V). The host fecundity cost of a virulent infection is measured by parameter *ψ*, and the additional host mortality rate induced by a virulent infection is measured by parameter *α*. Upon recovery from a virulent infection, individuals have a probability, θ, of gaining adaptive immunity (state R) which protects them from future infection costs. We shall refer to this as virulence immunity, to distinguish it from clinical immunity (used in the literature to refer to adaptive immunity to both severe and non-severe clinical malaria), or sterilising immunity (full immunity against infection itself, which does not occur for malaria [21]). Individuals who have gained virulence immunity can become infected and transmit the pathogen (state I) but these individuals do not suffer the deleterious effects of the infection. At this stage, individuals cycle between states R and I as they experience repeated infections and recoveries.

**Table 2:** The parameters of the model.

Parameter	Definition	Value used	Notes
*μ*	Death rate of hosts from causes other than malaria	1/30	mean lifespan of a host = 30 years
*σ*	Recovery rate from malaria infection	2	mean duration of infection = 6 months (mean durations of infection assumed for *P*. *falciparum* malaria in epidemiological models range from 20 to 200 days [87]; 6 months is in keeping with that assumed by [88], based on 20^th^ century malariatherapy studies.
β	Transmission parameter, related to the basic reproduction number (R_0_) in this model as follows: R_0_ = $\frac{\beta }{\sigma +\mu +\alpha }$	2.03–502	β values were chosen such that R_0_ takes values between 1 and 50.
*α*	Mortality rate due to malaria	Values between 0 and 10 tested.	The case fatality rate of malaria in this model is equal to $\frac{\alpha }{\sigma +\mu +\alpha }$. However, a more evolutionarily-relevant measure is the effect of *α* on the population overall, which depends on θ, σ, R_0_ and *r*. In the age structured model, when R_0_, as defined above = 5, *r* = 0.6 and σ = 2, if there is no virulence immunity (θ = 0) then a malaria mortality rate of α = 0.0075 means that each year the malaria deaths are equal to 15% of the births. However, if θ = 0.05, *α* = 0.0075 means that each year the malaria deaths are equal to 5% of the births, and if θ = 0.1, α = 0.0075 means malaria deaths each year are equal to 3% of the births.
*ψ*	Reproductive cost to the host of virulent infection with malaria	Values between 0 and 1 tested.	
*g*	Rate at which hosts become reproductively mature	∞ or 1/15	In the model without age structure, g is infinitely large. In the model including age structure, g = 1/15 and the mean time to reach reproductive maturity = 15 years.
*K*	Carrying capacity of population	10000	
*r*	Fecundity parameter, related to the birth rate as defined in Eq 10.	0.4 for the model without age structure or 0.6 for the model with age structure.	In the model including age structure, hosts are, on average, reproductively active for 2/3 of their lifespan. For better comparisons between Figs 2 and 3 we adjusted host fecundity to compensate for this, hence the two different values.
*c*_*i*_	Inherent fecundity cost of genotype *i*	For the wild type, *c*_*W*_ is always = 0; for the mutant genotype *c*_*M*_ may = 0.01 in Figs 2 and 3. We did not impose any inherent fecundity costs in the extended (three genotype) model, so c = 0.	
θ	Probability of a host gaining immunity to the virulent effects of malaria upon recovery from infection.	Values between 0 and 1 tested.	
*p*_*i*_	Proportion of infections blocked for genotype *i*	*p*_*W*_ (proportion of infections blocked for the wild type) = 0. In Figs 2–4, for the mutant genotype, *p*_*M*_ = 0.5. In Fig 5, the proportion of infections blocked by the FY*O homozygote (*p*_*hom*_) and the proportion of infections blocked by the FY*O heterozygote (*p*_*het*_) are varied. *p*_*het*_ is varied between 0 and 0.5, and *p*_*hom*_ is assumed to be 1.9 x *p*_*het*_.	
*q*_*i*_	Protection against malaria virulence enjoyed by genotype *i*.	For the wild type, *q*_*W*_ always = 0. In Figs 2–4, for the mutant genotype, *q*_*M*_ = 0. In Fig 5 *q*_*hom*_ and *q*_*het*_ are varied. *q*_*het*_ is varied between 0 and 0.5, and *q*_*hom*_ is assumed to be 1.9 x *q*_*het*_.	

We compartmentalised the host population into two genotypes: the wild type (W) and a mutant genotype which has evolved to fully or partially block infection (M). An infection being “blocked” means that a host who could have become infected is prevented from entering the V or I compartments—thus that host does not become infectious to others, and does not experience virulence. The blocking mutant therefore represents any adaptation which can reduce the chance of a malaria parasite establishing a blood stage infection (be it a mutation that blocks a parasite from entering a red blood cell, or a mutation that stops the entire infection process even earlier by blocking entry into a liver cell). We first focus on the outcome of competition between M and W genotypes, and hence for simplicity model a haploid population containing just these two genotypes. We later extend the model to simulate an evolving diploid human population. The bulk of *P*. *falciparum* virulence is borne by children. To allow for the possibility that immunity to malaria virulence may be gained before reproductive maturity, we further compartmentalised the host population into two reproductive states. Parameter *g* determines the transition rate between immature and mature states (represented by subscripts 1 and 2), where only mature hosts are able to reproduce.

We used the Next Generation approach [22,23] to analyse the invasion fitness of the mutant genotype under different conditions (see Methods). We also explored the speed of evolution (i.e. the rate at which we predict an infection blocking mutation would spread) using a model that tracks the joint epidemiological and evolutionary dynamics of a diploid host population.

## Results

### Adaptive immunity disadvantages infection blocking genotypes

**Fig 2:**
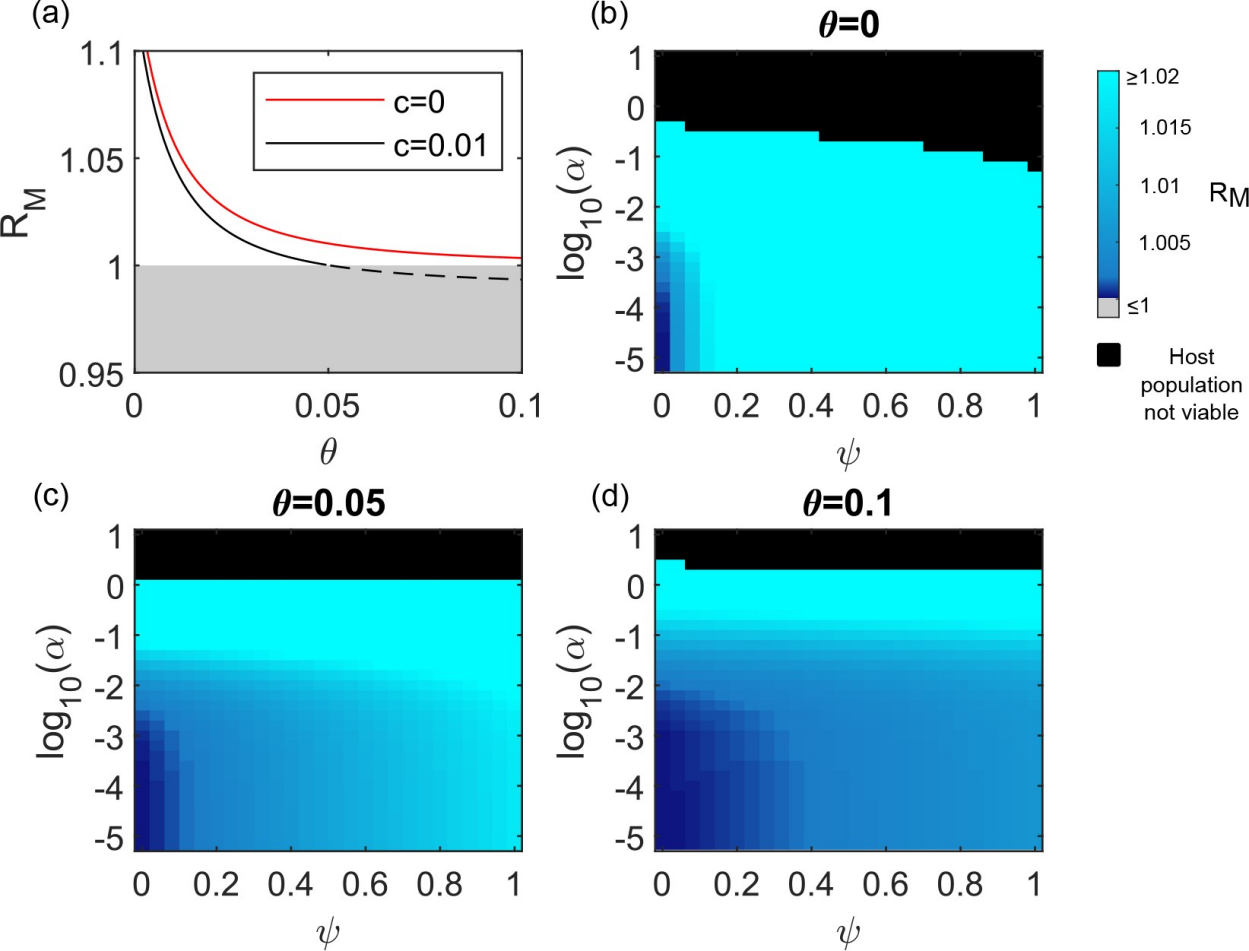
The success of infection blocking genotypes in the model without age structure. Panel (a) illustrates how R_M_ changes with varying values of θ (the probability of becoming immune to virulence upon recovery from infection) in the model without age structure. R_M_ must be > 1 if the infection blocking mutation is to spread. Values > 1 are indicated with a solid line and values ≤1 are indicated with a dashed line and a grey background. Models with and without an inherent cost to the infection blocking mutation (*c*≠0 and *c* = 0, respectively) are shown as indicated in the legend. The mutant genotype blocks 50% of infections (*p*_*M*_ = 0.5) and offers no other protection against virulence (*q*_*M*_ = 0). β = 10.2; *α* = 0.0075; *ψ* = 0.5 and other parameter values are as given in Table 2. Panels (b-d) illustrate the value of R_M_ for different combinations of the reproductive cost to the host of virulent infection (*ψ*) and the additional host mortality rate whilst virulently infected (*α*). The strength of virulence immunity (θ) increases with each panel (see panel titles). Black regions indicate that the equilibrium size of the resident wild type population is <1 and hence the host population is not viable. *c* = 0, β was varied between 10.1 and 60.1 so that R_0_ was kept at a value of 5, and all parameter values other than *ψ*_,_ *α* and θ are as described for panel (a).

First we consider a model without age structure. When the maturation rate *g* is infinite the hosts spend no time in the reproductively immature class. We introduce into this population a mutant genotype which halves the probability of a host becoming infected (*p*_*M*_ = 0.5). The per-generation invasion fitness of the mutant genotype (R_M_) decreases as the per-infection-probability of gaining virulence immunity (θ) increases (Fig 2A). If the infection blocking genotype is also associated with a small inherent fitness cost (*c* = 0.01), then above a threshold level of virulence immunity, R_M_ dips below 1 and the mutant genotype will not invade the population at all.

In the model without age structure, increasing either the mortality associated with pathogen virulence (*α*) or the host reproductive costs associated with pathogen virulence (*ψ*) increases R_M_ (Fig 2B–2D). This is the pattern we intuitively expect: the greater the costs of virulence, the greater the success of infection blocking genotypes. Virulence immunity reduces these costs, and hence reduces selection in favour of the infection blocking genotype.

### In an age structured model with adaptive immunity, increasing virulence can decrease the success of infection blocking mutations

**Fig 3:**
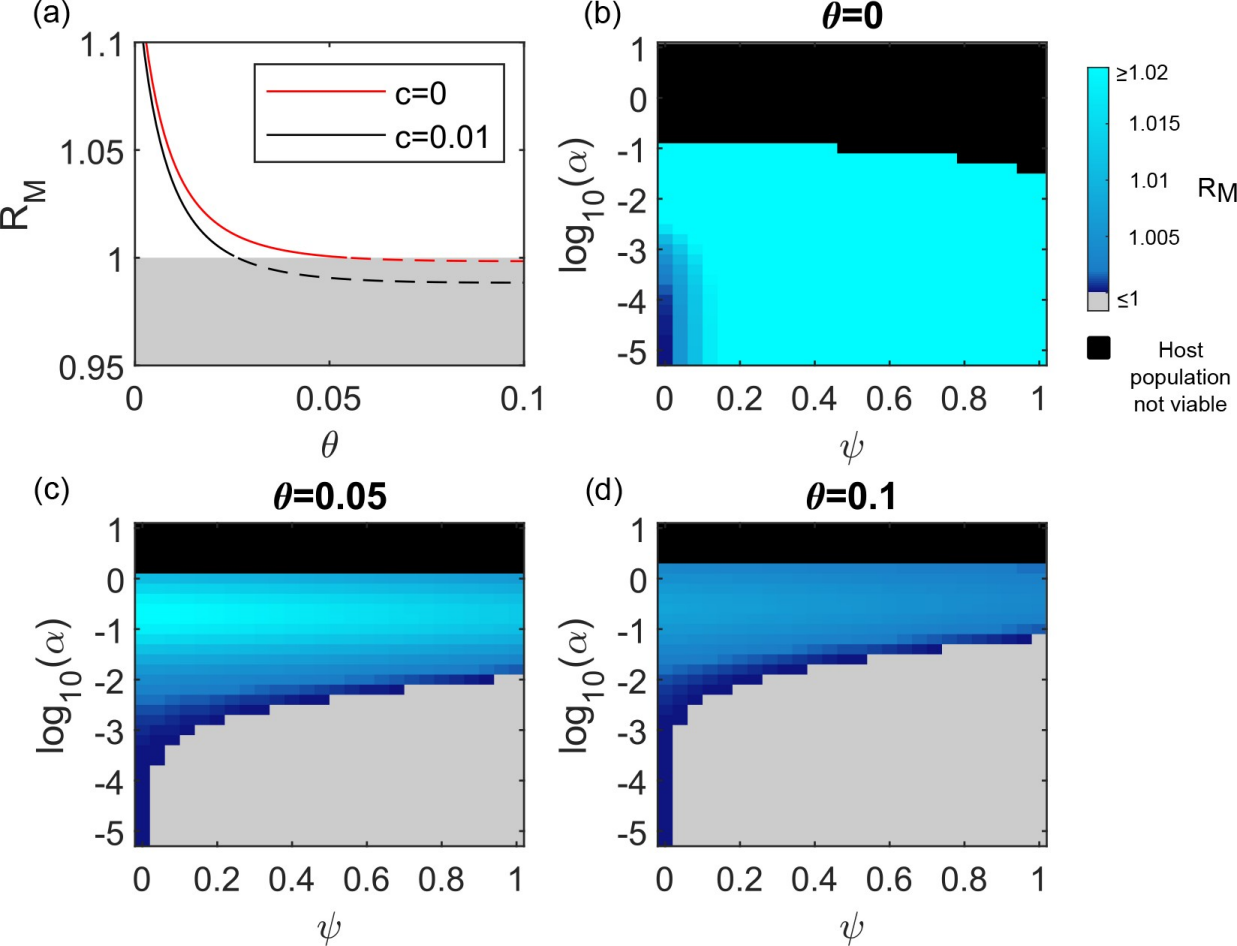
The success of infection blocking genotypes in the model including age structure. Panel (a) illustrates how R_M_ changes with varying values of θ (the probability of becoming immune to virulence upon recovery from infection) in the model including age structure. R_M_ must be > 1 if the infection blocking mutation is to spread. Values > 1 are indicated with a solid line and values ≤1 are indicated with a dashed line and a grey background. Models with and without an inherent cost to the infection blocking mutation (*c*≠0 and *c* = 0, respectively) are shown as indicated in the legend. The mutant genotype blocks 50% of infections (*p*_*M*_ = 0.5) and offers no other protection against virulence (*q*_*M*_ = 0). β = 10.2; *α* = 0.0075; *ψ* = 0.5 and other parameter values are as given in Table 2. Panels (b-d) illustrate the value of R_M_ for different combinations of the reproductive cost to the host of virulent infection (*ψ*) and the additional host mortality rate whilst virulently infected (*α*). The strength of virulence immunity (θ) increases with each panel (see panel titles). Grey regions indicate that R_M_ ≤ 1 (see colour bar). Black regions indicate that the equilibrium size of the resident wild type population is <1 and hence the host population is not viable. *c* = 0, β was varied between 10.1 and 60.1 so that R_0_ was kept at a value of 5, and all parameter values other than *ψ*_,_ *α* and θ are as described for panel (a).

We now consider a more realistic model in which hosts must age into a mature class in order to reproduce. We similarly introduce into this population a mutant genotype which halves the probability of a host becoming infected. The relationship between θ and R_M_ has the same shape as for the model without host aging (compare Fig 3A with Fig 2A). However, now that delayed reproduction is included, R_M_ dips below 1 as θ is increased even if the mutant genotype carries no inherent fitness cost (*c* = 0). Thus, in the age structured model, virulence immunity can prevent the invasion of a cost-free infection blocking mutant.

Counter-intuitively, as soon as virulence immunity is included in the age structured model, the relationship between R_M_ and the host reproductive cost of infection (*ψ*) is reversed. R_M_ now gets smaller as *ψ* gets larger (Fig 3C and 3D). A negative relationship between *ψ* and R_M_ arises because in the age structured model it is possible to gain virulence immunity prior to reaching reproductive maturity. Mutant genotype individuals experience fewer infections than wild type individuals, so have fewer opportunities to gain immunity whilst immature–leaving them more vulnerable to virulent infections whilst reproductively active. S1 Fig illustrates the expected times spent in different states for mutant and wild type genotypes. For the age structured model, above a threshold value of θ, the mutant genotype spends longer virulently infected whilst reproductively active (i.e. in class V_2_) than the wild type. This cannot occur in the model without age structure. If hosts experience a reproductive cost when virulently infected (*ψ*>0), spending longer virulently infected whilst reproductively active puts the mutant genotype at a disadvantage relative to the wild type host. The greater the reproductive cost of virulence, the greater this disadvantage, hence the negative relationship between *ψ* and R_M_.

If virulent infections carry mortality costs (α>0), the infection blocking mutant always has a longer average lifespan than the wild type. The higher the infection mortality rate, the bigger the discrepancy in life expectancy, and the greater the advantage to the mutant. There is, therefore, a largely positive relationship between α and R_M_ in the age structured model (Fig 3C and 3D). However, Fig 3C and 3D reveal a negative relationship between R_M_ and very high values of α in the presence of virulence immunity. Despite the infection blocking genotype always spending a longer *total* time in the reproductively mature class than the wild type, that gain in time as a proportion of the time spent reproductively mature by the wild type gets smaller at the highest values of α, if virulence immunity is present (S2 Fig). For a given probability of gaining virulence immunity (θ), the higher the value of α, the greater the proportion of individuals entering the mature class with virulence immunity. This is because higher values of α are more likely to quickly kill off individuals who have not yet gained virulence immunity. The greater the proportion of individuals entering the mature class with virulence immunity, the smaller the advantage to being an infection blocking mutant in the mature class. This means that above a certain level of α, increasing α starts to decrease the success of an infection blocking mutant, albeit never reducing the fitness of the infection blocking mutant to less than that of the wild type.

The combined effects of the two types of virulence (host reproductive costs, *ψ* and mortality costs, α) are essentially additive (Fig 3C and 3D). This means that as *ψ* is increased in the age structured model including virulence immunity, greater and greater values of α are necessary in order to allow an infection blocking mutation to spread at all.

### A low basic reproduction number for the parasite increases the success of infection blocking genotypes

**Fig 4:**
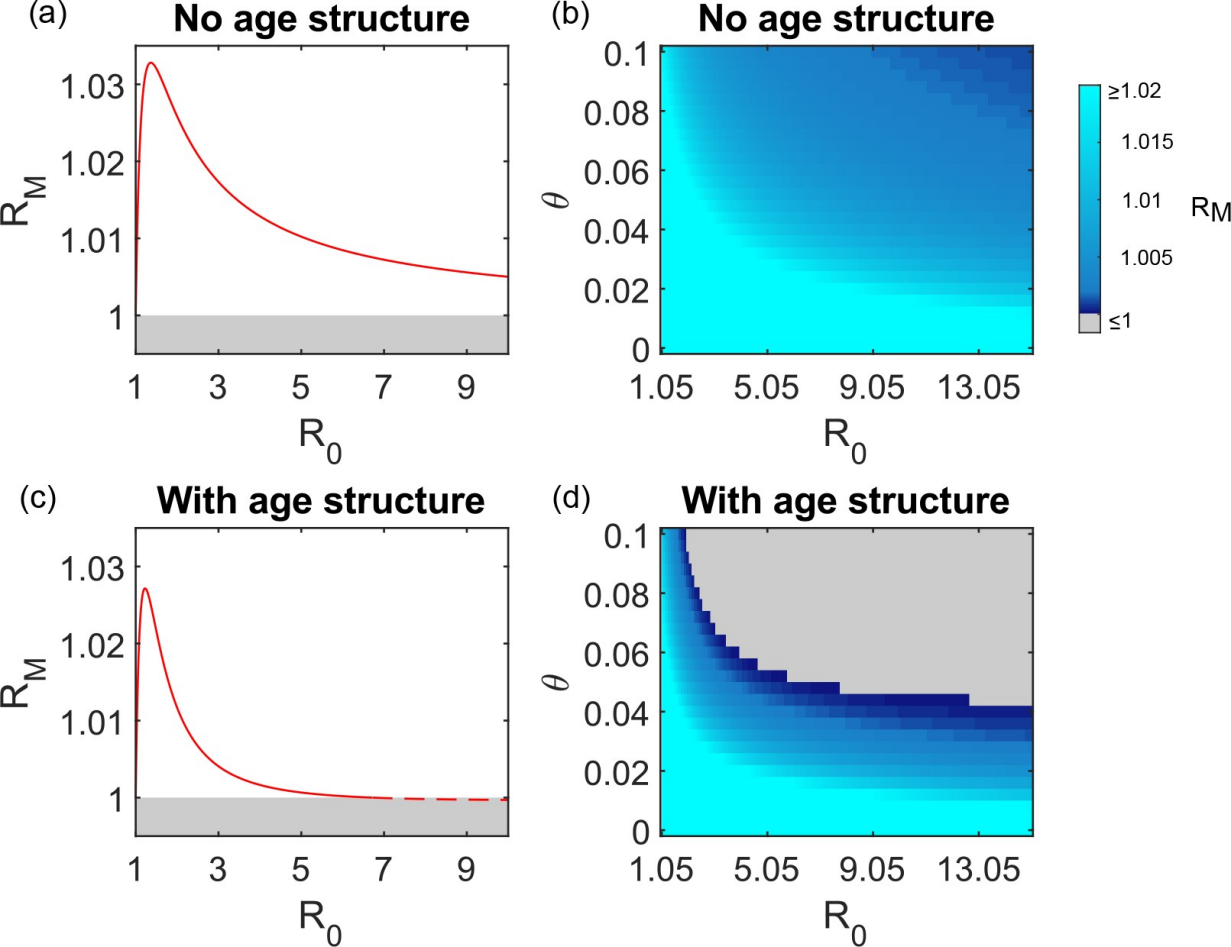
The impact of the basic reproduction number (R_0_) of the pathogen on the success of infection blocking genotypes. Panels (a) and (c) illustrates how R_M_ changes with varying values of R_0_ (here achieved by varying β –see Methods and Table 2). Panel (a) illustrates the model without age structure and panel (c) the model including age structure. Values of R_M_ > 1 are indicated with a solid line and R_M_ ≤1 is indicated with a dashed line. The mutant genotype blocks 50% of infections (*p*_*M*_ = 0.5) and offers no other protection against virulence (*q*_*M*_ = 0). θ = 0.05, *α* = 0.0075, *ψ* = 0.5 *c* = 0, and other parameter values are as given in Table 2. Panels (b) and (d) display R_M_ for different combinations of θ and R_0_. Panel (b) illustrates the model without age structure and panel (d) the model with age structure. Grey regions indicate that R_M_ ≤ 1 (see colour bar). All parameter values for panels (b) and (d) other than R_0_ and θ are as described for panels (a) and (c).

The basic reproduction number of the parasite (R_0_) also affects the success of infection blocking mutations. Fig 4 illustrates this for a mutant which blocks 50% of infections. R_M_ increases as R_0_ descends towards 1, reaching a peak at a low value of R_0_, before declining again to equal 1 when R_0_ equals 1. This behaviour occurs regardless of whether host age structure is included in the model (compare Fig 4A and 4C). Thus, at lower values of R_0_, infection blocking mutations can succeed in the age structured model even in the presence of strong virulence immunity (Fig 4D).

The overall difference in time spent virulently infected by the mutant genotype and the wild type genotype is maximised when R_0_ is low (S3 Fig). If infections are relatively rare, hosts of any genotype who recover from infection do not quickly become reinfected. Furthermore, when infections are infrequent, hosts of any genotype spend most of their lives without any adaptive immune protection against virulence immunity. Under these circumstances, being less likely to become infected in the first place creates a great advantage, hence R_M_ increases as R_0_ approaches 1. However, this advantage must be traded off against the fact that lower values of R_0_ reduce the total number of infections any host experiences in a lifetime. This means that R_M_ does not increase asymptotically as R_0_ approaches 1, and instead peaks at a value close to 1.

So far we have assumed that transmission is just as likely to occur from a non-virulent infection as a virulent infection, and that the background mortality rate of the immature class is the same as that of the mature class. Altering either of these assumptions does not change our overall conclusion that increasing the rate at which virulence immunity is gained (θ) decreases the success of infection blocking mutations (R_M_). The small changes to R_M_ which do occur when these assumptions are changed can be understood in terms of time spent virulently infected when reproductively mature. These results are explored in S1 Appendix and S4 and S5 Figs.

### A FY*O-like mutation can reach high frequencies in a human population within a realistic timeframe, provided virulence immunity is gained slowly or not at all

As noted in the introduction, we have one clear example of a malaria infection impeding mutation reaching high frequencies in human populations. Homozygosity for the Duffy negative (FY*O) mutation offers 49–69% protection against asymptomatic *P*. *vivax* infections in humans, and 96–98% protection against symptomatic infection [16]. FY*O rose to high frequencies in sub Saharan Africa within the last 34–49 thousand years [20]. To determine if our model can capture this behaviour, we extended the model to incorporate both heterozygotes and homozygotes for an infection blocking mutation (see Methods).

**Fig 5:**
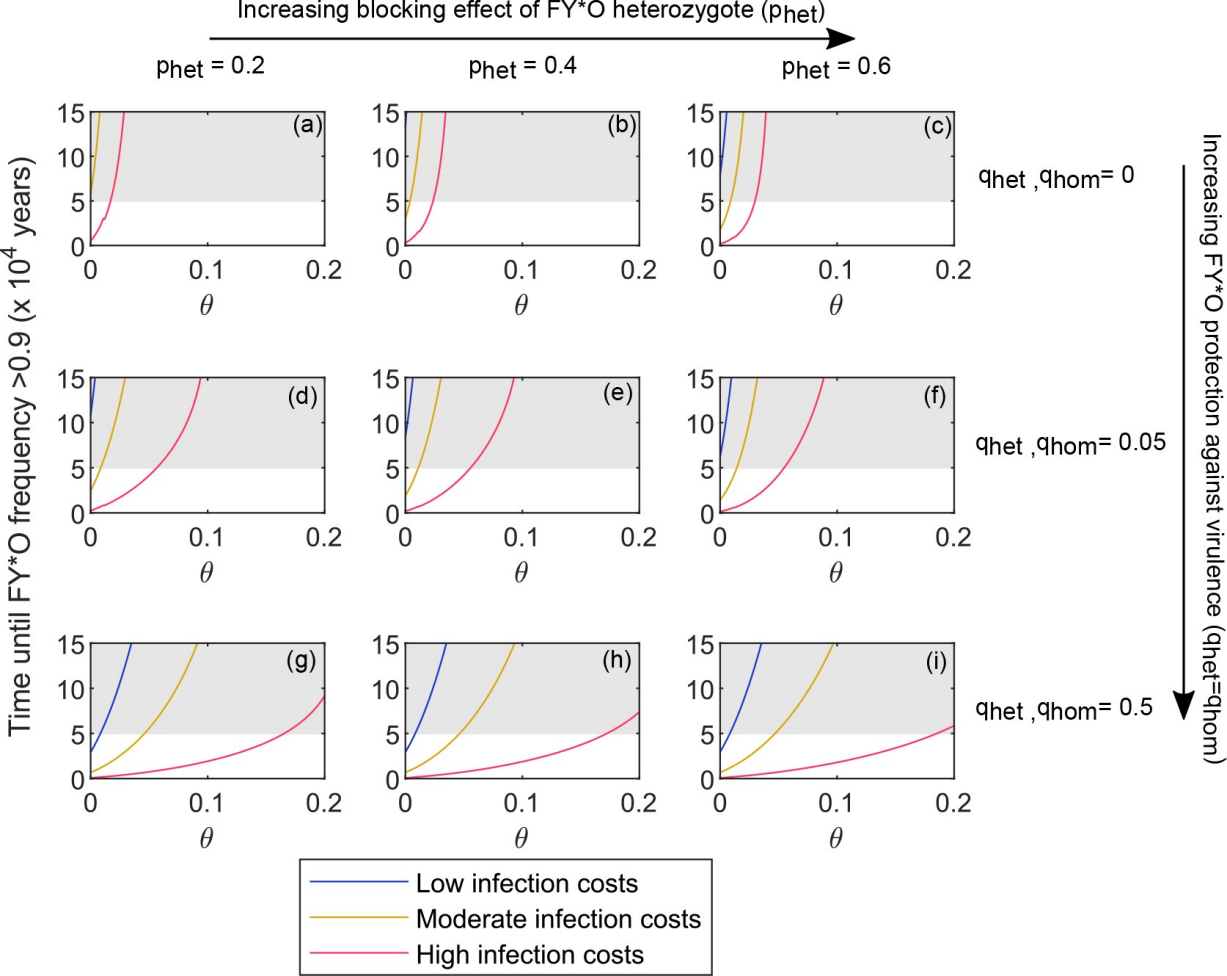
Time taken for FY*O to reach frequencies ≥ 90%. Panels (a-i) indicate the time taken for FY*O to reach a frequency ≥90% from a starting frequency of 0.1%, using the extended model (see Methods). We investigate different rates of gaining virulence immunity (θ, x axes), and each panel illustrates different possible properties of FY*O. The FY*O homozygote always blocks 96% of infections (p_hom_ = 0.96). From left to right across the figure, the infection blocking ability of the FY*O heterozygote increases (p_het_), and from the top to the bottom row of the figure the protection against virulence afforded by any genotype containing FY*O increases (q_het_ and q_hom_). Three different virulence scenarios have been included (see legend). In the low infection costs scenario, α = 0.0001 and ψ = 0.025; in the moderate infection costs scenario, α = 0.0005 and ψ = 0.1, and in the high infection costs scenario, α = 0.0075 and ψ = 0.5. The grey shaded region of each graph indicates unrealistic times (>49000 years). Other parameters were as listed in Table 2, or else were as follows: *g* = 1/15, *r* = 0.6, *c* = 0, β took values between 24.4 and 24.5 so as to keep R_0_ = 12.

Fig 5 illustrates the time FY*O takes to reach an allele frequency > 0.9 from a starting frequency of 0.1% [20] under different assumptions about the properties of FY*O; the level of selection from *P*. *vivax*, and the rate of gaining virulence immunity. Homozygosity for FY*O offers 96–98% protection against symptomatic infection, thus in all panels we assumed *p*_*hom*_ = 0.96. Changing this assumption so that *p*_*hom*_ = 49% (based on the level of protection Duffy negative individuals experience against asymptomatic infection) has little effect on the patterns seen (S6 Fig). Heterozygosity for FY*O may be protective against *P*. *vivax* infections [24], but the exact level of protection provided is unclear (Table 1). We therefore illustrate three different possible values of *p*_*het*_, increasing from left to right across Fig 5. It is possible that FY*O not only impedes *P*. *vivax* infection but also provides protection against virulence in any infection which does occur. For simplicity we assumed the same protection is provided to both heterozygotes and homozygotes (*q*_*hom*_ = *q*_*het*_). This means that protection against virulence is assumed to be a dominant trait. We varied this protection from top to bottom of Fig 5.

The faster the rate at which virulence immunity is gained (the higher the value of θ), the slower the spread of the FY*O mutation. If FY*O offers little or no protection against virulence (top two rows of Fig 5), we see a nonlinear relationship between θ and the time FY*O takes to spread, such that above a certain value of θ we essentially never expect FY*O to succeed. If FY*O offers strong protection against virulence (bottom row of Fig 5), then the relationship between θ and the time FY*O takes to spread becomes more linear, and allows for the possibility that FY*O could have spread within the last 49000 years at higher values of θ.

Fig 5 illustrates three different potential malaria infection costs scenarios (low, medium and high). Each has their own reproductive cost (ψ = 0.025, 0.1 or 0.5), which defines the probability of reproduction failing in a virulently infected host. If ψ = 0.5, virulently infected hosts reproduce at 50% of the rate of non virulently infected hosts. Each scenario also has its own malaria mortality rate (α = 0.0001, 0.0005 or 0.0075). These correspond to case fatality rates per individual malaria infection in those without virulence immunity of: 0.00005, 0.0002 and 0.0037 (see Table 2). The case fatality rate for *P*. *vivax* infection has been estimated at between 0.00012 and 0.00063 [25] in Papua New Guinea. If θ is low for *P*. *vivax* then the case fatality rate estimated by [25] directly corresponds to the case fatality rates for those without virulence immunity in our model, and the low and medium (blue and yellow) scenarios are the most plausible. However, if θ is high for *P*. *vivax* then many hosts in a population will have virulence immunity, so the case fatality rate for those without virulence immunity will be higher than the range reported in [25]. Under these circumstances, the high cost (red) scenario may be the most plausible. Under the low and medium cost scenarios, FY*O only reaches frequencies >0.9 within a realistic time frame if the rate of gaining virulence immunity is low (θ is close to zero). Under the high cost scenario, FY*O reaches frequencies >0.9 more quickly at values of θ close to zero, and FY*O must provide substantial additional protection against virulence in order to reach a frequency >0.9 at values of θ >0.05 (Fig 5I).

If it is possible to gain virulence immunity against *P*. *vivax* rapidly (e.g. θ >0.1), then we still observe that a higher R_0_ hinders the spread of a blocking mutation (S7 Fig). Higher values of R_0_ increase the time it takes for FY*O to reach frequencies >0.9.

## Discussion

We have demonstrated that the rate at which virulence immunity is gained has profound consequences for the evolution of mutations which block malaria infection. If virulent infection hinders the reproduction of the host, and if adaptive immunity protecting against such virulence is gained rapidly, a mutation whose major effect against malaria is to partially block infection will not succeed unless the force of infection is low. We noted in the introduction that the OK^-^ blood group; the Inab phenotype and the Lan null phenotype all have the potential to be *P*. *falciparum* blocking adaptations. However, none of these phenotypes have ever been reported at a high frequency in a malarious region. By contrast, a *P*. *vivax* blocking adaptation (FY*O) is present in malarious regions worldwide and has reached extremely high frequencies in sub Saharan Africa. The lack of success of the OK^-^ Inab and Lan null phenotypes in highly malarious regions can be explained by our model if the mutations responsible partly block *P*. *falciparum* infection in the heterozygous state, and immunity to *P*. *falciparum* virulence is gained rapidly (θ is high). The success of FY*O within a realistic timeframe is more likely if immunity to *P*. *vivax* virulence is gained slowly (θ is low). We propose that differences in the rate of gaining adaptive immunity to virulence caused by *P*. *falciparum* and *P*. *vivax* could account for the contrasting human adaptations to these two parasites.

The most compelling case for differences in the accumulation of immune responses to *P*. *falciparum* and *P*. *vivax* comes from a migration study [26]. Migrant workers and their families, who had not previously been exposed to malaria, moved to a malaria hyperendemic region of Indonesia. After just two years of the migrants living in the region, their susceptibility to malaria relative to the local residents was assessed. If immunity to malaria is built up cumulatively throughout life then the oldest local residents should display the greatest advantage against malaria relative to migrants of similar age, and the youngest local residents should have the least advantage against malaria relative to migrants of similar age. Exactly this pattern was observed for *P*. *vivax* parasitaemia, suggesting that immunity is indeed accumulated throughout life. However, local residents of any age group had a similar advantage against *falciparum* parasitaemia relative to the migrants. This indicated that >50 years of exposure to *P*. *falciparum* (as experienced by the oldest local residents) gave no additional advantage against *P*. *falciparum* than the ~3 years of exposure of the youngest local residents. Our model concerns virulence immunity, rather than immunity to parasitaemia *per se*, but [26] certainly indicates that some forms of *P*. *vivax* immunity are gained cumulatively over the course of many infections, whilst *P*. *falciparum* immunity is gained more rapidly.

Further evidence supporting a rapid gain of *P*. *falciparum* virulence immunity is the fact that in high transmission regions, severe *P*. *falciparum* malaria syndromes (other than pregnancy associated malaria) are concentrated in children below five years of age [27]. It is possible that immunity to non-cerebral severe malaria disease is gained after just one or two infections [28]. Parameter θ in our model may therefore be >0.1 for *P*. *falciparum*—i.e. fewer than 10 infections may be required to elicit *P*. *falciparum* virulence immunity. Our results show that such a value of θ could drastically slow, or stop the spread of *P*. *falciparum* infection blocking mutations in a range of settings (Figs 3 and 4).

Understanding virulence immunity in *P*. *vivax* is challenging, because there is no part of the world where *P*. *vivax* circulates alone, meaning co-infection with *falciparum* is always a potential confounding factor. Children in regions where *vivax* and *falciparum* co-circulate seem to become immune to fevers caused by *vivax* more rapidly than they become immune to fevers caused by *falciparum* [29]. This observation contradicts our proposal that immunity to *vivax* virulence may be gained slowly. However, it is not clear that immunity to fever corresponds to immunity to all the costs of infection. Two studies in Indonesia and West Papua have found severe malaria cases associated with either *P*. *vivax* or *P*. *falciparum* to be concentrated in younger age groups [7,8], suggesting little difference in the rate at which virulence immunity is gained for the two parasites. However, a separate study in Indonesia [6] showed that older individuals remain at risk from serious disease caused by *P*. *vivax*, whereas the risk for serious disease caused by *P*. *falciparum* was concentrated in younger age groups. This observation suggests slower or less reliable acquisition of virulence immunity in *P*. *vivax*.

A crucial assumption of our model is that malaria generates a reproductive cost for the host. Allowing immunity gained in childhood to protect against a later reproductive cost is the main mechanism by which adaptive immunity can prevent the spread of infection blocking mutations. Reproductive costs exist for both *P*. *falciparum* and *P*. *vivax* infection in humans [30,31]. Pregnant women seem to be especially susceptible to infection with either *P*. *falciparum* or *P*. *vivax*, in particular during their first pregnancies [30]. Infection during pregnancy can lead to low birth weights, miscarriage, stillbirth, and maternal mortality. It is also clear that immunity gained prior to becoming pregnant can mitigate the reproductive costs of *P*. *falciparum* (outlined in the next paragraph), but relevant studies are lacking for *P*. *vivax*.

White [32] points out a critical difference in *P*. *falciparum* infection outcomes during pregnancy according to the prior immune experience of the mother. In regions of high transmission, where mothers will have gained immunity prior to becoming pregnant, the main cost of malaria in pregnancy is low birth weight, caused by placental malaria. In low transmission regions, where mothers have little or no malaria immunity, pregnant women additionally experience severe malaria syndromes, with maternal death, premature labour and stillbirth all potential outcomes [32]. The association between *P*. *falciparum* and stillbirth is greater in lower transmission (i.e. less immune) regions [33]. Temporary infertility following episodes of *P*. *falciparum* malaria has been observed in a non-immune adult man [34], thus *P*. *falciparum* infection in the absence of virulence immunity may have reproductive costs for men also. Overall, the assumption in our model that exposure to malaria in childhood can protect against particularly bad reproductive outcomes in adulthood is reasonable for *P*. *falciparum*.

Is there any evidence that immunity to bad reproductive outcomes is gained more quickly for *P*. *falciparum* as opposed to *P*. *vivax*? If immune responses can mitigate the reproductive costs of malaria, we expect costs to be on average highest in first pregnancies, and smaller in subsequent pregnancies (assuming that time approximates exposure to malaria). In a generally low-immune setting (Thailand), *P*. *falciparum* infection during pregnancy was associated with a greater decrease in birth weight during first pregnancies than in later pregnancies [35]–in keeping with protective immunity to *P*. *falciparum* building up over time. However, *P*. *vivax* infection during pregnancy was associated with a greater decrease in birthweight in *later* pregnancies than in first pregnancies [35], which supports the possibility that immunity to that particular form of reproductive virulence caused by *P*. *vivax* is not gained rapidly.

What sort of biological mechanism could explain a fundamental difference in virulence immunity between *P*. *falciparum* and *P*. *vivax*? Antigens eliciting virulence immunity of the type we have modelled would have to (i) be variable (so that more than one exposure is required in order to build up immunity against different types), and (ii) encode proteins involved in disease severity (so that immunity against this particular antigen directly protects against virulence). The most well-studied such antigen for *P*. *falciparum* is *P*. *falciparum* erythrocyte protein 1 (Pfemp1), a highly variable antigen encoded by the multigene *var* family. Pfemp1 mediates the cytoadherence of infected red blood cells to other red blood cells (rosetting) or to endothelial surfaces, both of which are strongly implicated in disease severity. A somewhat similar multigene family in *P*. *vivax* is *vir* [36]. Individuals immune to *P*. *vivax* have differing profiles of reactivity to *vir* variants (presumably reflecting their history of infection). Evidence is growing that *P*. *vivax* can cytoadhere (albeit to a lower extent than *P*. *falciparum*) and *vir* gene products may be involved [37,38]. The different biological properties of *var* and *vir* could account for differing rates of gaining virulence immunity to *P*. *falciparum* and *P*. *vivax* respectively. *var* genes are expressed clonally: only one variant of pfemp1 is expressed on the erythrocyte surface at any one time. By contrast, multiple different *vir* genes are expressed simultaneously in cells infected by *P*. *vivax* [39]. Clonal expression of Pfemp1 means that theoretically a single antibody response against a specific variant of Pfemp1 protects against virulence mediated by that Pfemp1. Thus, if certain Pfemp1 variants are associated with the worst outcomes in severe *P*. *falciparum* malaria, virulence immunity can be gained quickly because only a few different antibody responses against those specific Pfemp1 variants are required. It is indeed the case that only a subset of Pfemp1 variants are associated with severe malaria [40,41], and the variants associated with severe disease are more likely to be expressed in immunologically naïve hosts [41]. For non clonally expressed *vir* genes, an antibody response against just one *vir* is unlikely to provide substantial protection against damaging cytoadherence, because cytoadherence could be mediated by so many different *vir* proteins simultaneously. Thus, immunity to a virulence effect mediated by the *vir* gene family is likely to be gained much more slowly. We are still far from a full understanding of virulence in either *P*. *falciparum* or *P*. *vivax*, so a direct comparison between *var* and *vir* may prove inappropriate, but the contrast between the two does illustrate one reason why virulence immunity to *P*. *falciparum* and *P*. *vivax* might not be gained at the same rate.

Slowly acquired virulence immunity to *P*. *vivax* is not the only possible explanation for the success of FY*O offered by our model. The red scenario in Fig 5 corresponds to a case fatality rate of 0.0037 for *P*. *vivax* infection in a person without virulence immunity, alongside a 50% reduction in reproductive success for hosts whilst they are virulently infected. If these costs are plausible for *P*. *vivax*, and if FY*O offers some inherent protection against those costs, as well as blocking infections, then the FY*O mutation can spread even if virulence immunity is gained relatively quickly (Fig 5I). *P*. *vivax* has both reproductive and mortality costs, but quantifying them is challenging. The odds ratio for *P*. *vivax* infection as a risk factor for miscarriage, for example, ranges between 0.65 and 4.64 in different studies [30]. One study estimated the case fatality rate for *P*. *vivax* in Papua New Guinea at between 0.00012 and 0.00063 by combining hospital surveillance with community surveillance and questionnaires about treatment seeking behaviour[25], but this is likely to underestimate the historical case fatality rate. Furthermore, the case fatality rate which can be calculated for our model applies only to infections in those who have not gained virulence immunity, whereas the case fatality rate calculated by [25] averages across their estimate of the total number of *P*. *vivax* infections. Without knowing the rate of gaining *P*. *vivax* virulence immunity, it is impossible to fully determine the correct mortality rate to apply to virulent *P*. *vivax* infection within our framework.

Roche et al [42] recently argued that the observed costs of *P*. *vivax* to humans are not sufficient to account for the success of FY*O. Instead, co-infection between *P*. *vivax* and *P*. *falciparum*, which has especially poor outcomes [8], might have made it advantageous for a *P*. *vivax* blocking mutation to spread. This hypothesis is also able to account for the fact that FY*O only reaches near-fixation in sub Saharan Africa, despite the presence of *P*. *vivax* elsewhere in the world: a lower transmission potential of *P*. *falciparum* outside of sub Saharan Africa reduces selection in favour of FY*O. Our model does not contradict the Roche hypothesis. The important point is not the exact mechanism by which the cost arises–rather, how easy it is to gain immunity against the cost. If *vivax* and *falciparum* co-infection does cause humans a heavy cost, and it is hard to gain immunity against that cost, our model shows this will promote the success of FY*O. When it comes to the question of why FY*O has succeeded in sub Saharan Africa rather than in Asia, the most obvious explanation offered by our model is that the R_0_ of *P*. *vivax* might have been lower in sub Saharan Africa than in Asia (since within our model, the advantage to a blocking mutation increases at lower values of R_0_). We cannot rule this out, but present-day temperature evidence suggests that conditions for *P*. *vivax* transmission are similar in both regions[43]. A deeper understanding of the history of *P*. *vivax* adaptation at the FY locus will require the expansion of our model to allow for (i) *P*. *falciparum* and *P*. *vivax* co-infection, and (ii) the existence of alleles encoding different Duffy proteins as well as the null allele at the Duffy locus (see Table 1). Competition between other FY variants, as well as FY*O, will have contributed to the pattern we see today.

Far greater genetic sampling of populations in sub Saharan Africa is required before we can be confident that there is not a widespread human *P*. *falciparum* blocking adaptation. However, we can be relatively sure that no *P*. *falciparum* blocking mutation has reached the frequencies observed for FY*O in sub Saharan Africa, since no population displays the resistance to *P*. *falciparum* that FY*O affords against *P*. *vivax*. Some data are available regarding the three candidate loci we previously described, where *P*. *falciparum* blocking adaptations might be expected to be driven to high frequencies by natural selection (Basigin, CD55 and ABCB6). Some CD55 amino acid substitutions occur at elevated frequencies in malarious regions, the highest of which is A227P in 14.43% of the Luhya in Webuye population in Kenya [18]. However, this mutation would not lead to the Inab phenotype, which is caused by missense or splice site altering mutations, and its blocking effect is unknown. Some Lan null causing mutations in ABCB6 occur at significantly higher frequencies in malaria exposed populations than in non-malaria exposed populations, but the reverse is also true for different Lan null causing mutations [19]. The highest allele frequency of a Lan null causing mutation reported in [19] is 0.0154 in African populations. This is an aggregated African population, since finer scale surveys of different global populations are not available. Attempts to find a link between severe malaria outcomes and CD55 [44] or basigin [45] associated SNPs have so far failed [44,45].

We predict that mutations whose only effect is to partially block malaria infection should be most successful in regions where the R_0_ of malaria is closely above 1 (Fig 4). It would therefore be particularly interesting to conduct population genetic surveys in regions where *P*. *falciparum* transmission was historically low or intermittent, and determine whether OK^-^, Lan null, or Inab phenotype mutations occur at elevated frequencies.

We can rule out two alternative hypotheses as to why FY*O succeeded where *P*. *falciparum* blocking mutations failed. Fitness costs of *P*. *falciparum* blocking mutations are unlikely to be responsible since one OK^-^ woman is noted to have had 5 children [46], and the Inab phenotype has been reported in a 91 year old Japanese woman [47] and an 86 year old Italian American woman and her 70 year old brother [48]. Some Inab phenotype individuals suffer gastrointestinal problems but others do not [49]. The Lan null phenotype is only clinically relevant for blood transfusions or rare instances of foetal-maternal incompatibility of blood type [50]. It is also unlikely that the FY*O mutation arises more frequently than mutations which could block *P*. *falciparum* infection. FY*O always involves exactly the same promoter region mutation (T>C at position -33). By contrast, the Inab phenotype has been shown to be caused by at least three different mutations [49], and the Lan null phenotype by at least ten [50].

There are, however, other potential explanations for humanity’s differing adaptations to *P*. *falciparum* and *P*. *vivax*, which do not necessarily require the mechanisms we propose here. There is an inherent stochasticity in the success or failure of individual mutations, so it does remain possible that the lack of a *P*. *falciparum* blocking adaptation is due to chance alone. It is also likely that humans have co-evolved with *P*. *vivax* for longer than *P*. *falciparum* [51], so we cannot rule out the possibility that there has not yet been long enough for one of the *P*. *falciparum* blocking adaptations spread successfully in a highly malarious zone.

*P*. *vivax* can enter a dormant liver state and give rise to recrudescent infections. A single infection with *P*. *vivax* can thus cause several risky episodes of parasitaemia. Does this mean that there is a greater selective pressure to block an initial infection with *P*. *vivax*, as opposed to blocking “singular” *P*. *falciparum* infections? We have not explicitly included a dormant stage in our model. However, we varied the costs of virulent infection over a wide range. When it comes to the fitness of the host overall, there is little difference between three separate episodes of parasitaemia, or one episode with three times the cost. Furthermore, so long as there is a chance of building up virulence immunity over the course of repeated episodes of parasitaemia, then regardless of whether those episodes come from new infections or recrudescence, we still predict that the higher the rate of gaining virulence immunity, the slower the spread of infection blocking mutations. Nevertheless, there may be additional implications of a dormant stage which we need to fully address in a future version of the model.

A final alternative explanation for different adaptations to *P*. *falciparum* and *P*. *vivax* is epistasis between malaria protective mutations. We have previously shown that epistasis amongst globin gene mutations can explain the relative absence of the highly malaria protective sickle cell mutation from certain malarious regions [52,53]. The existence of certain adaptations to *P*. *falciparum* in a population may preclude the evolution of infection blocking adaptations. Future modelling, with a greater focus on the ability of mutations to prevent virulence (parameter q in our model) will shed more light on this possibility.

## Conclusions

The absence of *P*. *falciparum* infection blocking adaptations from humans could lead us to assume that mutations which limit the entry of *P*. *falciparum* to human cells carry high costs. By including adaptive immunity in an evolutionary-epidemiological model, we have shown this does not have to be the case. Instead, adaptive immunity itself can make infection blocking a disadvantageous strategy. Human mutations which partially block malaria infection in the heterozygous state, but do not offer any additional protection against malaria virulence, will only succeed under very limited circumstances. Either (i) anti-virulence immunity must take very many repeated infections to arise, or (ii) the basic reproductive number of malaria must be not far above 1, so that transmission can take place, but hosts only become infected at a relatively low rate.

Within our framework, the global rarity of blood types such as OK^-^, Lan null and the Inab phenotype is an inevitable consequence of the rapid acquisition of *P*. *falciparum* virulence immunity. We predict that mutations with the potential to fully block *P*. *falciparum* in the homozygous state are most likely to be found in populations living at the very limits of malaria transmission. The success of the Duffy null mutation implies the existence of virulent effects of *P*. *vivax* to which immunity is gained only slowly or not at all. The impact of *P*. *vivax* on birth weight is a key candidate for such an effect. Overall, the markedly different evolutionary strategies by which humans have adapted to *P*. *falciparum* and *P*. *vivax* seem likely to reflect fundamental differences in our immunological responses to each parasite. Our observations underscore the need for further studies to unpick the interplay between immunity and virulence for both parasite species.

## Methods

### The model

Eqs 1–8 describe the rates of change of numbers of immature and mature susceptible (S_1_ and S_2_), virulently infected (V_1_ and V_2_), resistant to virulence (R_1_ and R_2_) and infectious but not at risk of excess mortality (I_1_ and I_2_) individuals of genotype *i*.

$$\frac{dS_{1i}}{dt}=b_{i}+(1-\theta )\sigma V_{1i}-\left((1-p_{i})\lambda +\mu +g\right)S_{1i}$$ Eq 1

$$\frac{dV_{1i}}{dt}=(1-p_{i})\lambda S_{1i}-(\sigma +\mu +(1-q_{i})\alpha +g)V_{1i}$$ Eq 2

$$\frac{dR_{1i}}{dt}=\theta \sigma V_{1i}+\sigma I_{1i}-\left((1-p_{i})\lambda +\mu +g\right)R_{1i}$$ Eq 3

$$\frac{dI_{1i}}{dt}=(1-p_{i})\lambda R_{1i}-(\sigma +\mu +g)I_{1i}$$ Eq 4

$$\frac{dS_{2i}}{dt}=gS_{1i}+(1-\theta )\sigma V_{2i}-((1-p_{i})\lambda +\mu )S_{2i}$$ Eq 5

$$\frac{dV_{2i}}{dt}=gV_{1i}+(1-p_{i})\lambda S_{2i}-(\sigma +\mu +(1-q_{i})\alpha )V_{2i}$$ Eq 6

$$\frac{dR_{2i}}{dt}=gR_{1i}+\theta \sigma V_{2i}+\sigma I_{2i}-((1-p_{i})\lambda +\mu )R_{2i}$$ Eq 7

$$\frac{dI_{2i}}{dt}=gI_{1i}+(1-p_{i})\lambda R_{2i}-(\sigma +\mu )I_{2i}$$ Eq 8

The force of infection, *λ*, is given by Eq 9 where *N* = the total population size. The birth rate, *b*_*i*_ is given by Eq 10, where *K* = the carrying capacity of the population; *r* = a fecundity parameter *c*_*i*_ captures any inherent fecundity cost of genotype *i*, and *ψ* determines the host reproductive cost of virulent infection. Values used for these parameters are given in Table 2.

$$\lambda =\frac{\beta \sum_{i=0}^{2}(V_{1i}+I_{1i}{+V}_{2i}+I_{2i})}{N}$$ Eq 9

$$b_{i}=r.(1-c_{i})\left(S_{2i}+R_{2i}+I_{2i}+{(1-(1-q_{i})\psi )V}_{2i}\right)\left(1-\frac{N}{K}\right)$$ Eq 10

Parameter *θ* controls the proportion of hosts recovering out of compartment *V* who gain adaptive immunity protecting them against death from infection. *θ* therefore represents the probability of gaining protective immunity when a host recovers from infection, which means that the average number of infections experienced before protective immunity is achieved is equal to 1/*θ*. Parameter *p*_*i*_ controls the protection against infection afforded to genotype *i*. If *p*_*i*_ = 1, genotype *i* is fully protected against infection; if *p*_*i*_ = 0, genotype *i* becomes infected at the maximum possible rate within the system. Parameter *q*_*i*_ controls the protection against the virulent effects of malaria afforded to genotype *i*. If *q*_*i*_ = 1, genotype *i* is fully protected against all virulence; if *q*_*i*_ = 0, genotype *i* dies whilst infected at the maximum possible rate within the system and suffers the maximum reproductive cost of virulence. Other parameters of the model and the values used in our analyses are described in Table 2.

### Analysing the model

We wished to determine the circumstances under which a mutant host genotype can successfully invade a population consisting entirely of a wild type (resident) host genotype. The Next Generation Theorem [22,54]offers an elegant method to solve evolutionary invasion problems of this sort (e.g. [23]). To apply this method, we represented the different states of our mutant population as a vector, *H*_*M*_.

*H*_*M*_ = (*S*_1*M*_ *V*_1*M*_ *I*_1*M*_ *R*_1*M*_ *S*_2*M*_ *V*_2*M*_ *I*_2*M*_ *R*_2*M*_)^*T*^. The matrix *A*_*M*_ contains the growth rates of the mutant genotype in each of those states, such that $\frac{dH_{M}}{dt}=A_{M}H_{M}$. The matrix *A*_*M*_ can be decomposed into two further matrices, *V*_*M*_ and *F*_*M*_, related to *A*_*M*_ as follows: *A*_*M*_ = *F*_*M*_−*V*_*M*_.

*F*_*M*_ contains all the terms which capture the processes by which new mutant genotype individuals are introduced into the system, and thus captures fecundity. *V*_*M*_ describes the transitions between different host states. *A*_*M*_, *F*_*M*_ and *V*_*M*_ for our model are given in the supplementary material (S1 Appendix).

According to the Next Generation Theorem, if all elements of *F*_*M*_ and $V_{M}^{-1}$ are greater than or equal to zero (true for our system), then the mutant genotype will spread in the population if the dominant eigenvalue of $F_{M}V_{M}^{-1}$ is greater than 1. Henceforth ${B_{M}=F}_{M}V_{M}^{-1}$. The dominant eigenvalue of *B*_*M*_ can be conceptualised as the average lifetime reproductive output of an individual of the mutant genotype. We shall represent this quantity as *R*_*M*_. *R*_*M*_ must be greater than 1 in order for a single mutant genotype individual to spread in the resident population.

The main model we present is not a fully accurate representation of human reproduction (reproduction in the main model occurs clonally), but *R*_*M*_ can still be interpreted as representing the reproductive success of a single new mutant genotype individual entering a population of the resident host. In an extended model (see next section) we include both heterozygotes and homozygotes for the novel mutation, to explore how the introduced mutation increases in frequency in the population over time.

We wished to determine whether a mutant genotype can invade a resident wild type host population where the parasite is already at its endemic equilibrium. For any given set of parameters we first solved the system numerically in the absence of the mutant genotype to obtain values for *N* (the equilibrium population size of the resident host population), and *λ* (the force of infection with the parasite at equilibrium). To obtain these numerical solutions we used solver ode15s in Matlab with the following options: relative error tolerance of 1x10^-10^, absolute error tolerance of 1x10^-12^, and the ‘nonnegative’ option included. We then used those numerically-derived values of *N* and *λ*, together with the other parameters, to calculate *R*_*M*_ for any given set of parameter values. Matlab was used to perform these calculations.

### The extended model

The model described above is designed to determine whether the mutant can spread upon initially arriving in a wild type population, but does not address how a mutant allele changes in frequency in a human population over time. In order to explore this, we split the host population into three genotypes: the wild type (subscript 1), heterozygotes for the infection blocking mutation (subscript 2), and homozygotes for the infection blocking mutation (subscript 3). Each of these genotypes is associated with an infection blocking property (*p*_*1*_, *p*_*2*_ and *p*_*3*_, where *p*_*1*_ always = 0), and with a certain degree of protection against malaria virulence (*q*_*1*_, *q*_*2*_ and *q*_*3*_, where *q*_*1*_ always = 0). Eqs 11–13 capture how these effects were introduced in the birth terms of the model (replacing Eq 10), and Eq 14 calculates the allele frequency of the mutation, which feeds into Eqs 11–13. Eqs 1–9 still apply, apart from the fact that the number of host genotypes in this extended model is 3, not 2.

$$b_{1}=r\left(\sum_{i=1}^{3}(S_{2i}{+I}_{2i}+R_{2i}+{(1-(1-q_{i})\psi )V}_{2i})\right){\left(1-a\right)}^{2}\left(1-\frac{N}{K}\right)$$ Eq 11

$$b_{2}=r\left(\sum_{i=1}^{3}\left(S_{2i}{+I}_{2i}+R_{2i}+{(1-(1-q_{i})\psi )V}_{2i}\right)\right)2(1-a)a\left(1-\frac{N}{K}\right)$$ Eq 12

$$b_{3}=r\left(\sum_{i=1}^{3}\left(S_{2i}{+I}_{2i}+R_{2i}+{(1-(1-q_{i})\psi )V}_{2i}\right)\right)a^{2}\left(1-\frac{N}{K}\right)$$ Eq 13

$$a=\frac{S_{2,3}{+I}_{2,3}+R_{2,3}+{(1-(1-q_{3})\psi )V}_{2,3}+0.5(S_{2,2}{+I}_{2,2}+R_{2,2}+{(1-(1-q_{2})\psi )V}_{2,2})}{\sum_{i=1}^{3}\left(S_{2i}{+I}_{2i}+R_{2i}+{(1-(1-q_{i})\psi )V}_{2i}\right)}$$ Eq 14

## Supporting information

S1 Appendix Methodological details and supplementary results. This appendix consists of 3 parts: 1. The F_M_ and V_M_ matrices for the models used in the main text (pages 2–3); 2. Mechanisms underlying the patterns observed in the main text (pages 4–5), and 3. Investigating variable transmission rates and variable background mortality (pages 6–8).
(PDF)

S1 Fig The impact of adaptive immunity (θ) on the time hosts spend in reproductively active classes. Solid lines indicate the time spent in each class by the mutant genotype and dashed lines indicate the time spent in each class by the resident (wild type) genotype. For details of how the time spent in each class is calculated, please see S1 Appendix, section 2. Panel (a) illustrates the model without age structure and panel (b) the model including age structure. Parameters were as follows: *μ* = 1/30; *g* = 1/15; *σ* = 10; *α* = 0.05; *λ* = 5; *q*_*M*_ = 0; *p*_*M*_ = 0.5; *c* = 0.
(PDF)

S2 Fig The impact of infection mortality (α) on the time spent immature and mature in the age structured model. A logarithmic scale has been used for α in order to facilitate comparison with Fig 3 of the main text. The upper panels show the expected times in each class. For details of how the time spent in each class is calculated, please see S1 Appendix, section 2. Solid lines indicate the mutant genotype and dashed lines indicate the resident (wild type) genotype. The lower panels show the difference between the time the mutant spends in a class and the time the wild type spends in a class, as a proportion of the time the wild type spends. Column (a) illustrates the age structured model in the absence of adaptive immunity (θ = 0) and column (b) includes adaptive immunity (θ = 0.01). Other parameters were as follows: *μ* = 1/30; *g* = 1/15; *σ* = 10; *λ* = 5; *q*_*M*_ = 0; *p*_*M*_ = 0.5; *c* = 0.
(PDF)

S3 Fig The relationship between R_0_, R_M_ and time spent virulently infected (V) whilst reproductively active. At low values of R_0_, the mutant tends to spend less time virulently infected than the wild type, regardless of whether or not age structure is included in the model (top panels of S3 Fig). For details of how the time spent in each class is calculated, please see S1 Appendix, section 2. There is a value of R_0_ close to 1 which maximises the difference in time spent. These differences in times spent virulently infected whilst reproductively active account for the behaviour of R_M_ at values close to 1 (compare top and bottom panels of S3 Fig). Parameters used were as follows: = 1/30; *g* = 1/15; *σ* = 10; *q*_*M*_ = 0; *p*_*M*_ = 0.5 θ = 0.05; α = 0; ψ = 1; *c* = 0. To aid comparison with Fig 4, the x axis displays values of R_0_ (but note that in order to emphasise the effect of time spent virulently infected whilst reproductively mature we have used ψ = 1 and α = 0, whilst Fig 4 uses ψ = 0.1 and α = 0.01). The transition matrix (from which we obtained the times spent in each class) requires the force of infection (*λ*). For each value of R_0_ we used numerical simulations to identify the equilibrium proportion of infected individuals in the absence of the mutation, and from this obtained an appropriate value of *λ* for each R_0_ value.
(PDF)

S4 Fig Altering our assumption of uniform background mortality does not affect the overall relationship between the rate of gaining virulence immunity (θ) and the success of blocking mutations (R_M_). Panel (a) illustrates the effect of varying θ on R_M_ under three different background mortality scenarios, using the supplementary model (S1 text section 3). The black line is equivalent to the scenario shown in Fig 4A of the main text, where immature and mature hosts both experience the same background mortality (μ_1_ = μ_2_ = 1/30). The blue line shows the case where the immature class experiences a higher background mortality than the mature (μ_1_ = 1/15; μ_2_ = 1/30). The red shows the case where the immature class experiences a lower background mortality than the mature (μ_1_ = 0; μ_2_ = 1/30). Other parameters were as follows: β_V_ = β_N_ = 10.2; *g* = 1/15; *σ* = 2; *q*_*M*_ = 0; *p*_*M*_ = 0.5; α = 0.0075; ψ = 0.5; *c* = 0. Panel (b) illustrates the difference in average time spent reproductively mature between the mutant and the wild type (time spent by mutant minus time spent by wild type) for the different scenarios illustrated in panel (a). For details of how the time spent in each class is calculated, please see S1 Appendix, section 2. Panel (c) illustrates the difference in the time spent virulently infected whilst reproductively mature (class V_2_) between the mutant and the wild type (time spent by mutant minus time spent by wild type) for the different scenarios illustrated in panel (a). The colours of the lines in panels (b) and (c) have the same meanings as those in panel (a).
(PDF)

S5 Fig Altering our assumption of uniform transmission from virulence and mild infection does not affect the overall relationship between the rate of gaining virulence immunity (θ) and the success of blocking mutations (R_M_). We illustrate the effect of varying θ on R_M_ under three different infection transmission scenarios, using the supplementary model (see S1 text section 3). The black line is equivalent to the scenario shown in Fig 4A of the main text, where both virulent infections and non-virulent infections transmit at the same rate (β_V_ and β_N_ = 10.20). The blue line shows the case where virulent infections transmit more than non-virulent infections (β_V_ = 30.6 and β_N_ = 10.20). The red line illustrates a scenario where non-virulent infections transmit more than virulent infections (β_V_ = 10.20 and β_N_ = 30.6). Other parameters were as follows: μ_*1*_ = 1/30; *μ*_*2*_ = 1/30; *g* = 1/15; *σ* = 2; *q*_*M*_ = 0; *p*_*M*_ = 0.5; α = 0.0075; ψ = 0.5; *c* = 0.
(PDF)

S6 Fig Time taken for FY*O to reach frequencies ≥ 90%, assuming a lower blocking ability of the FY*O homozygote. Panels (a-i) indicate the time taken for FY*O to reach a frequency ≥90% from a starting frequency of 0.1%, using the extended model (see Methods). We investigate different rates of gaining virulence immunity (θ, x axes), and different properties of FY*O. We assume the FY*O homozygote blocks 59% of infections (p_hom_ = 0.59). From left to right across the figure, the infection blocking ability of the FY*O heterozygote increases (p_het_), and from the top to the bottom row of the figure the protection against virulence afforded by any genotype containing FY*O increases (q_het_ and q_hom_). Three different virulence scenarios have been included (see legend). In the low infection costs scenario, α = 0.0001 and ψ = 0.025; in the moderate infection costs scenario, α = 0.0005 and ψ = 0.1, and in the high infection costs scenario, α = 0.0075 and ψ = 0.5. The grey shaded region of each graph indicates unrealistic times (>49000 years). Other parameters were as listed in Table 2, or else were as follows: *g* = 1/15, *r* = 0.6, *c* = 0, β took values between 24.4 and 24.5 so as to keep R_0_ = 12.
(PDF)

S7 Fig Time taken for FY*O to reach frequencies ≥ 90%, for different values of R_0_. Panels (a-c) indicate the time taken for FY*O to reach a frequency ≥90% from a starting frequency of 0.1%, using the extended model (see Methods). We investigate different rates of gaining virulence immunity (θ, x axes), and different virulence protection properties of FY*O. From the top to the bottom row of the figure the protection against virulence afforded by any genotype containing FY*O increases (q_het_ and q_hom_). In all panels, the FY*O heterozygote blocks 40% of infections (p_het_ = 0.4), and the FY*O homozygote blocks 96% of infections (p_hom_ = 0.96). Results are shown for three different values of R_0_ for malaria (see legend). The grey shaded region of each graph indicates unrealistic times (>49000 years). Other parameters were as listed in Table 2, or were as follows: α = 0.0075, ψ = 0.5, *g* = 1/15, *r* = 0.6, *c* = 0, β took values between 24.4 and 24.5 so as to generate the necessary values of R_0_.
(PDF)
