# Supplementary material for: Adaptive immunity selects against malaria infection blocking mutations
Source: PLoS Comput Biol. 2020 Oct 8;16(10):e1008181. doi: 10.1371/journal.pcbi.1008181 (PMC7544067; doi:10.1371/journal.pcbi.1008181)
Supplement: S1 Appendix — This appendix consists of 3 parts: 1. The FM and VM matrices for the models used in the main text (pages 2–3); 2. Mechanisms underlying the patterns observed in the main text (pages 4–5), and 3. Investigating variable transmission rates and variable background mortality (pages 6–8). (PDF) [file pcbi.1008181.s001.pdf]

Adaptive immunity selects against malaria infection blocking mutations

Appendix S1: Methodological details and supplementary results.

This appendix consists of 3 sections:

1. The F_M and V_M matrices for the models used in the main text (pages 2-3)

2. Mechanisms underlying the patterns observed in the main text (pages 4-5)

3. Investigating variable transmission rates and variable background mortality (6-8)

1. The F_M and V_M matrices for the models used in the main text.

The different states of the mutant population (susceptible, virulently infected, etc.) can be represented as a vector, H_M. The matrix A_M contains the growth rates of the mutant genotype in each of those states, such that $\frac{dH_M}{dt} = A_M H_M$. A_M can be decomposed into two further matrices, V_M and F_M, related to A_M as follows: $A_M = F_M - V_M$. F_M captures host fecundity and V_M describes the transitions between different host states. F_M and V_M for our model with and without host aging are given below.

1.1 F_M and V_M in the simple model (without host aging)

Here, H_M=[S,V,R,I]^T.

$$F_M = \begin{pmatrix} r - \frac{rN}{K} & (1-(1-q_M)\psi)\left(r - \frac{rN}{K}\right) & r - \frac{rN}{K} & r - \frac{rN}{K} \\ 0 & 0 & 0 & 0 \\ 0 & 0 & 0 & 0 \\ 0 & 0 & 0 & 0 \\ 0 & 0 & 0 & 0 \end{pmatrix}$$

$$V_M = \begin{pmatrix} \mu + \lambda(1-p_M) & -\sigma(1-\theta) & 0 & 0 \\ -\lambda(1-p_M) & \mu + \sigma + \alpha(1-qM) & 0 & 0 \\ 0 & -\theta\sigma & \mu + \lambda(1-p_M) & -\sigma \\ 0 & 0 & -\lambda(1-p_M) & \mu + \sigma \end{pmatrix}$$

1.2 F_M and V_M in the model including host aging

Here, H_M=[S_1,V_1,R_1,I_1, S_2,V_2,R_2,I_2]^T, where subscript 1 indicates the immature state and subscript 2 indicates the reproductively mature state.

$$F_M = \begin{pmatrix} 0 & 0 & 0 & 0 & r- \frac{rN}{K} & (1-(1-q_M)\psi)\left(r - \frac{rN}{K}\right) & r- \frac{rN}{K} & r- \frac{rN}{K} \\ 0 & 0 & 0 & 0 & 0 & 0 & 0 & 0 \\ 0 & 0 & 0 & 0 & 0 & 0 & 0 & 0 \\ 0 & 0 & 0 & 0 & 0 & 0 & 0 & 0 \\ 0 & 0 & 0 & 0 & 0 & 0 & 0 & 0 \\ 0 & 0 & 0 & 0 & 0 & 0 & 0 & 0 \\ 0 & 0 & 0 & 0 & 0 & 0 & 0 & 0 \\ 0 & 0 & 0 & 0 & 0 & 0 & 0 & 0 \end{pmatrix}$$

$$
V_M = \begin{pmatrix}
g+\mu+\lambda(1-p_M) & -\sigma(1-\theta) & 0 & 0 & 0 & 0 & 0 & 0 \\
-\lambda(1-p_M) & g+\mu+\alpha(1-q_M)+\sigma & 0 & 0 & 0 & 0 & 0 & 0 \\
0 & -\theta\sigma & g+\mu+\lambda(1-p_M) & -\sigma & 0 & 0 & 0 & 0 \\
0 & 0 & -\lambda(1-p_M) & g+\mu+\sigma & 0 & 0 & 0 & 0 \\
-g & 0 & 0 & 0 & \mu+\lambda(1-p_M) & -\sigma(1-\theta) & 0 & 0 \\
0 & -g & 0 & 0 & -\lambda(1-p_M) & \mu+\alpha(1-q_M)+\sigma & 0 & 0 \\
0 & 0 & -g & 0 & 0 & -\theta\sigma & \mu+\lambda(1-p_M) & -\sigma \\
0 & 0 & 0 & -g & 0 & 0 & -\lambda(1-p_M) & \mu+\sigma
\end{pmatrix}
$$

2. Mechanisms underlying the patterns observed in the main text.

R_M is the average number of offspring an individual of the infection-blocking mutant genotype (M) will contribute to the population upon first entering a population of the resident host (W). R_M can be expressed as the sum of the expected fecundity of the different classes of the mutant host:

$$R_M = \sum_i F_{M,i} T_{M,i}$$

where $F_{M,i}$ is the fecundity rate of mutant class *i* (see expression for F_M in section 1) and $T_{M,i}$ is the expected time spent in that class. The first column of the inverse of V_M gives the expected time a newborn of the mutant genotype spends in each model class. The first column of the inverse of V_W (identical to V_M other than that p_W=0 and q_W = 0) gives the expected time a newborn of the wild type genotype spends in each class.

$F_{M,i}$ depends on the properties of each class of hosts, and on how far the resident population is from its carrying capacity (K-N).

R_M can also be expressed as the ratio of the reproductive potential of the mutant genotype to that of the resident host:

$$R_M = \frac{\sum_i B_{M,i} T_{M,i}}{\sum_i B_{W,i} T_{W,i}}.$$

where $B_{M,i}$ is the inherent reproductive potential of mutant class *i* (independent of N) ; $B_{W,i}$ is the inherent reproductive potential of resident host class *i* and $T_{k,i}$ is the expected time host type *k* spends spent in class *i*.

In the model without age structure, hosts exist in 4 different classes: S,V,R and I (susceptible, virulently infected, recovered and infected), all of which can reproduce. In the model including age

structure hosts exist in 8 different classes but only 4 have nonzero fecundity rates: S_2, V_2, R_2 and I_2 (susceptible and mature; virulently infected and mature; recovered and mature, and infected and mature). Figures S1-S3 (see separate files) illustrate the times the mutant genotype and the wild type host spend in different reproductively active states as specific parameters are varied. R_M is determined by the times spent in these states, underlying the phenomena observed in figures 2, 3 and 4 of the main text.

3. Investigating variable transmission rates and variable background mortality.

3.1.1 Methods

In the model presented in the main text, we assumed that hosts experiencing virulent infections and hosts experiencing non-virulent infections were equally infectious. We also assumed that the background death rate was constant in both the reproductively immature and the reproductively mature age classes. To understand whether changing either of these assumptions affect our overall conclusions, we produced a supplementary version of the model in which the force of infection (λ) allowed virulent and non-virulent infections to have different transmission rates (equation S1). We also allowed the background mortality rate (μ) to take two different values: μ_1 for all reproductively immature states and μ_2 for all reproductively mature states.

$$\lambda = \frac{\beta_V \sum_{i=0}^{2}(V_{1i}+V_{2i}) + \beta_N \sum_{i=0}^{2}(I_{1i} + I_{2i})}{N} \qquad \text{Equation S1}$$

2.1.2 Results

Figure S4 examines the effect of changing the relative background mortalities of the immature and mature classes. Changing the background mortality rate of each class does not alter our overall conclusion that increasing the rate at which virulence immunity is gained (θ) decreases the success of infection blocking mutations (R_M), as illustrated in S4a.

When there is no virulence immunity (θ=0), having the immature age class experience lower background mortality increases R_M relative to the scenario shown in the main text (figure S4a inset: the red line is higher than the black line). Similarly, when there is no virulence immunity, having the immature age class experience higher background mortality decreases R_M relative to the scenario shown in the main text (the blue line is lower than the black line). This can be explained by considering the overall amount of time which the mutant gets to spend in the reproductively active

age class (figure S4b, see section 2 of this appendix for how these times are calculated). The mutant genotype always spends longer than the wild type in the reproductively active age class, because the mutant genotype experiences fewer infections overall, so is less likely to be killed by one of those infections. However, when the immature age class dies at a lower background rate (red line), and virulence immunity is not possible (θ=0), this difference is at its most pronounced. When the background mortality rate of the immature age class is low, the main reason that a host will not reach reproductive maturity is if they are killed by the infection. The mutant genotype can block such infections. The bigger the impact of infection on whether or not a host reaches reproductive maturity (i.e. the bigger the proportion of immature age class mortality that is attributable to infection), the greater the advantage to the mutant genotype, and the higher the value of R_M.

As the rate of gaining virulence immunity increases (θ >0), we observe a switch such that having the immature age class experience lower mortality leads to lower R_M values than in the scenario in the main text (the red line moves below the black line in figure S4a). To understand this, we need to consider the trade-off between the overall time spent reproductively active *and* the time spent virulently infected once reproductively mature. Figure S4c illustrates that as θ increases, the mutant genotype spends relatively more time virulently infected whilst reproductively mature (i.e. in class V_2) than the wild type. This puts the mutant genotype at a disadvantage, reducing R_M. The magnitude of this effect is greatest when the immature age class experiences a lower mortality rate (the red line reaches the highest values in figure S4c). This effect arises because when the immature class has a lower mortality rate, more of those surviving to adulthood will have achieved virulence immunity before they reach adulthood. The more adults there are who do not experience virulent infection, the lower the advantage to the mutant genotype.

Overall, reducing the mortality of the immature age class will (i) increase the overall time that hosts spend reproductively mature, and (ii) increase the proportion of mature hosts who are

immune to virulence (if such immunity is possible). The relative advantage to the mutant genotype of the combination of these processes depends on the rate at which virulence immunity is gained.

Figure S5 examines the effect of allowing either higher transmission from virulent infections or higher transmission from non-virulent infections, relative to the baseline case of the same transmission from both. Altering the pattern of transmission from the different types of infection does not change our overall conclusion that increasing the rate at which virulence immunity is gained (θ) decreases the success of infection blocking mutations (R_M).

Allowing extra transmission from either type of infection slightly reduces R_M relative to the baseline case (the red and blue lines fall below the black line in figure S4). If virulence immunity is not possible (θ=0), increasing transmission from non-virulent infection makes no difference, but increasing transmission from virulent infections causes a large reduction in R_M. As θ increases, increasing transmission from either virulent or non-virulent infection generates more and more similar reductions in R_M, until above a certain value of θ increasing transmission from non-virulent infection causes a slightly greater reduction in R_M. In the main text we report that increasing R_0 reduces R_M (figure 4 and figure S3). The patterns seen in figure S4 arise through the same mechanism. The more that the pathogen is transmitted (by whatever route), the more likely it is that a host will gain virulence immunity before reaching reproductive maturity, and hence the lower the advantage to the infection blocking mutation. The relative importance of transmission from either virulent or non-virulent infections changes according to the proportion of each in the population, which is determined by θ.
