# Supplementary material for: Adaptive immunity selects against malaria infection blocking mutations
Source: PLoS Comput Biol. 2020 Oct 8;16(10):e1008181. doi: 10.1371/journal.pcbi.1008181 (PMC7544067; doi:10.1371/journal.pcbi.1008181)
Supplement: S1 Fig — Solid lines indicate the time spent in each class by the mutant genotype and dashed lines indicate the time spent in each class by the resident (wild type) genotype. For details of how the time spent in each class is calculated, please see S1 Appendix, section 2. Panel (a) illustrates the model without age structure and panel (b) the model including age structure. Parameters were as follows: μ = 1/30; g = 1/15; σ = 10; α = 0.05; λ = 5; qM = 0; pM = 0.5; c = 0. (PDF) [file pcbi.1008181.s002.pdf]

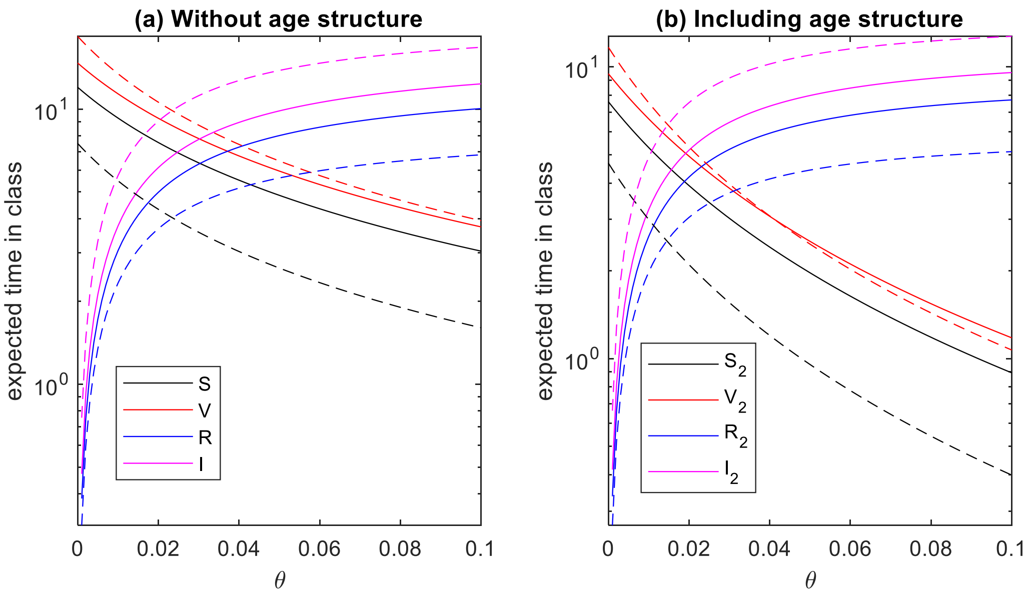

Figure S1: The impact of adaptive immunity (θ) on the time hosts spend in reproductively active classes. Solid lines indicate the time spent in each class by the mutant genotype and dashed lines indicate the time spent in each class by the resident (wild type) genotype. For details of how the time spent in each class is calculated, please see Appendix S1, section 2. Panel (a) illustrates the model without age structure and panel (b) the model including age structure. Parameters were as follows: μ= 1/30; g=1/15; σ=2; α=0.0075; λ=5; q_M=0; p_M=0.5; c=0.
