# Supplementary material for: Adaptive immunity selects against malaria infection blocking mutations
Source: PLoS Comput Biol. 2020 Oct 8;16(10):e1008181. doi: 10.1371/journal.pcbi.1008181 (PMC7544067; doi:10.1371/journal.pcbi.1008181)
Supplement: S2 Fig — A logarithmic scale has been used for α in order to facilitate comparison with Fig 3 of the main text. The upper panels show the expected times in each class. For details of how the time spent in each class is calculated, please see S1 Appendix, section 2. Solid lines indicate the mutant genotype and dashed lines indicate the resident (wild type) genotype. The lower panels show the difference between the time the mutant spends in a class and the time the wild type spends in a class, as a proportion of the time the wild type spends. Column (a) illustrates the age structured model in the absence of adaptive immunity (θ = 0) and column (b) includes adaptive immunity (θ = 0.01). Other parameters were as follows: μ = 1/30; g = 1/15; σ = 10; λ = 5; qM = 0; pM = 0.5; c = 0. (PDF) [file pcbi.1008181.s003.pdf]

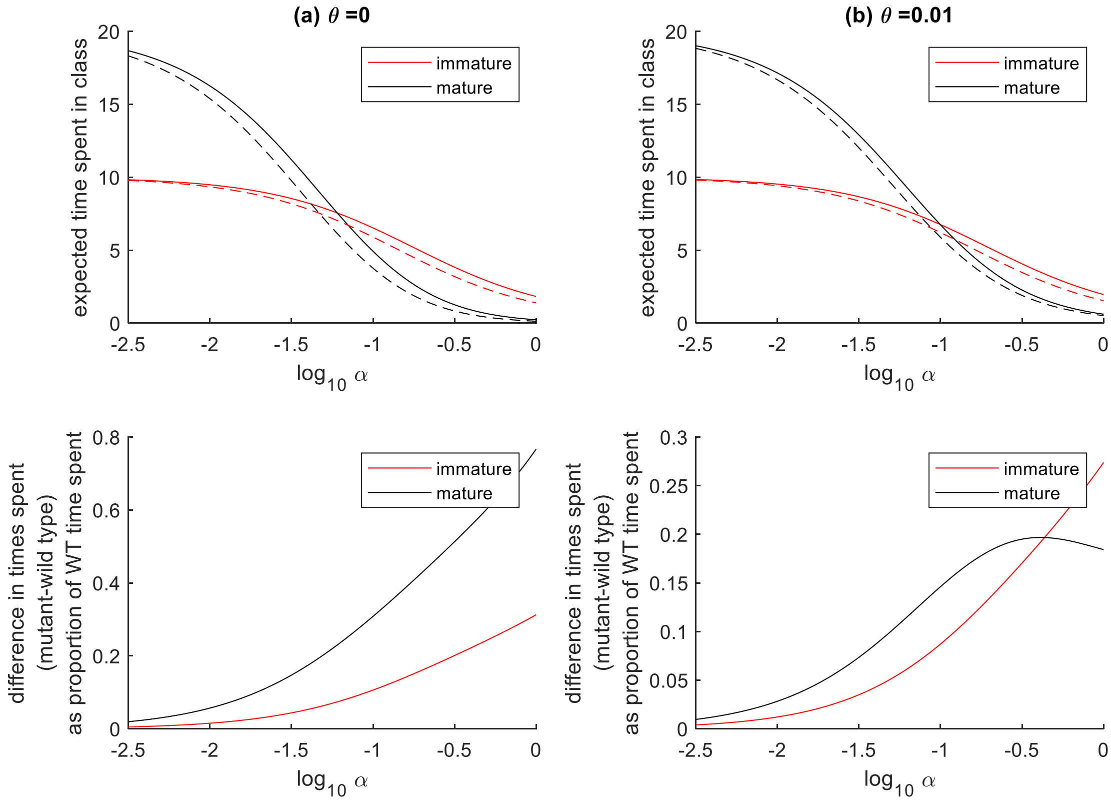

Figure S2: The impact of infection mortality (α) on the time spent immature and mature in the age structured model. A logarithmic scale has been used for α in order to facilitate comparison with figure 3 of the main text. The upper panels show the expected times in each class. For details of how the time spent in each class is calculated, please see Appendix S1, section 2. Solid lines indicate the mutant genotype and dashed lines indicate the resident (wild type) genotype. The lower panels show the difference between the time the mutant spends in a class and the time the wild type spends in a class, as a proportion of the time the wild type spends. Column (a) illustrates the age structured model in the absence of adaptive immunity (θ=0) and column (b) includes adaptive immunity (θ=0.01). Other parameters were as follows: μ= 1/30; g=1/15; σ=2; λ=5; q_M=0; p_M=0.5; c=0.
