# Supplementary material for: Adaptive immunity selects against malaria infection blocking mutations
Source: PLoS Comput Biol. 2020 Oct 8;16(10):e1008181. doi: 10.1371/journal.pcbi.1008181 (PMC7544067; doi:10.1371/journal.pcbi.1008181)
Supplement: S3 Fig — At low values of R0, the mutant tends to spend less time virulently infected than the wild type, regardless of whether or not age structure is included in the model (top panels of S3 Fig). For details of how the time spent in each class is calculated, please see S1 Appendix, section 2. There is a value of R0 close to 1 which maximises the difference in time spent. These differences in times spent virulently infected whilst reproductively active account for the behaviour of RM at values close to 1 (compare top and bottom panels of S3 Fig). Parameters used were as follows: = 1/30; g = 1/15; σ = 10; qM = 0; pM = 0.5 θ = 0.05; α = 0; ψ = 1; c = 0. To aid comparison with Fig 4, the x axis displays values of R0 (but note that in order to emphasise the effect of time spent virulently infected whilst reproductively mature we have used ψ = 1 and α = 0, whilst Fig 4 uses ψ = 0.1 and α = 0.01). The transition matrix (from which we obtained the times spent in each class) requires the force of infection (λ). For each value of R0 we used numerical simulations to identify the equilibrium proportion of infected individuals in the absence of the mutation, and from this obtained an appropriate value of λ for each R0 value. (PDF) [file pcbi.1008181.s004.pdf]

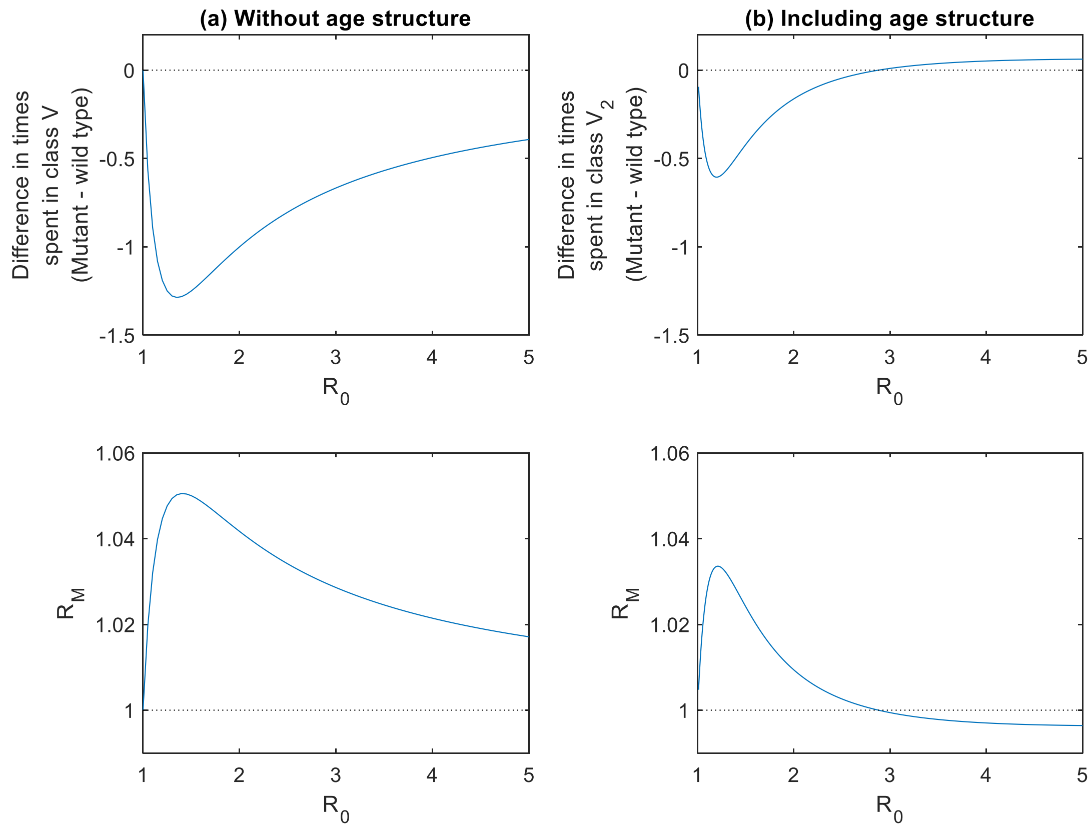

Figure S3: The relationship between R_0, R_M and time spent virulently infected (V) whilst reproductively active. At low values of R_0, the mutant tends to spend less time virulently infected than the wild type, regardless of whether or not age structure is included in the model (top panels of figure S3). There is a value of R_0 close to 1 which maximises the difference in time spent. For details of how the time spent in each class is calculated, please see Appendix S1, section 2. These differences in times spent virulently infected whilst reproductively active account for the behaviour of R_M at values close to 1 (compare top and bottom panels of figure S3). Parameters used were as follows: = 1/30; g=1/15; σ=2; q_M=0; p_M=0.5 θ=0.05; α=0; ψ=1; c=0. To aid comparison with figure 4, the x axis displays values of R_0 (but note that in order to emphasise the effect of time spent virulently infected whilst reproductively mature we have used ψ=1 and α=0, whilst figure 4 uses ψ=0.5 and α=0.0075). The transition matrix (from which we obtained the times spent in each class) requires the force of infection (λ). For each value of R_0 we used numerical simulations to identify the equilibrium proportion of infected individuals in the absence of the mutation, and from this obtained an appropriate value of λ for each R_0 value.
