# Supplementary material for: Adaptive immunity selects against malaria infection blocking mutations
Source: PLoS Comput Biol. 2020 Oct 8;16(10):e1008181. doi: 10.1371/journal.pcbi.1008181 (PMC7544067; doi:10.1371/journal.pcbi.1008181)
Supplement: S4 Fig — Panel (a) illustrates the effect of varying θ on RM under three different background mortality scenarios, using the supplementary model (S1 text section 3). The black line is equivalent to the scenario shown in Fig 4A of the main text, where immature and mature hosts both experience the same background mortality (μ1 = μ2 = 1/30). The blue line shows the case where the immature class experiences a higher background mortality than the mature (μ1 = 1/15; μ2 = 1/30). The red shows the case where the immature class experiences a lower background mortality than the mature (μ1 = 0; μ2 = 1/30). Other parameters were as follows: βV = βN = 10.2; g = 1/15; σ = 2; qM = 0; pM = 0.5; α = 0.0075; ψ = 0.5; c = 0. Panel (b) illustrates the difference in average time spent reproductively mature between the mutant and the wild type (time spent by mutant minus time spent by wild type) for the different scenarios illustrated in panel (a). For details of how the time spent in each class is calculated, please see S1 Appendix, section 2. Panel (c) illustrates the difference in the time spent virulently infected whilst reproductively mature (class V2) between the mutant and the wild type (time spent by mutant minus time spent by wild type) for the different scenarios illustrated in panel (a). The colours of the lines in panels (b) and (c) have the same meanings as those in panel (a). (PDF) [file pcbi.1008181.s005.pdf]

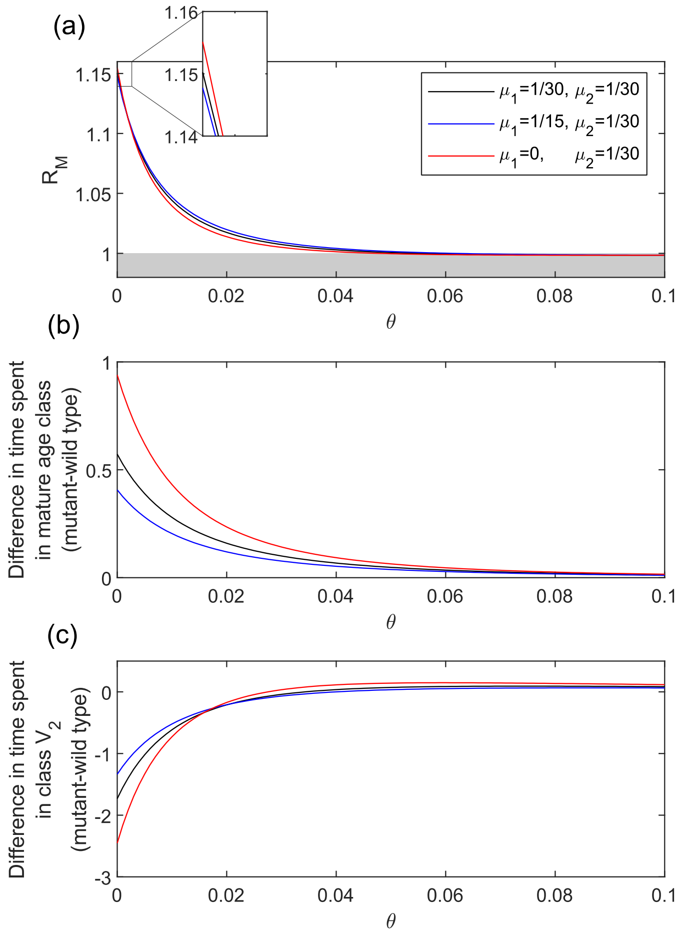

Figure S4: Altering our assumption of uniform background mortality does not affect the overall relationship between the rate of gaining virulence immunity (θ) and the success of blocking mutations (R_M). Panel (a) illustrates the effect of varying θ on R_M under three different background mortality scenarios, using the supplementary model (S1 text section 3). The black line is equivalent to the scenario shown in figure 4a of the main text, where immature and mature hosts both experience the same background mortality ($\mu_1 = \mu_2 = 1/30$). The blue line shows the case where the immature class experiences a higher background mortality than the mature ($\mu_1 = 1/15$; $\mu_2 = 1/30$). The red shows the case where the immature class experiences a lower background mortality than the mature ($\mu_1 = 0$; $\mu_2 = 1/30$). Other parameters were as follows: $\beta_V = \beta_N = 10.2$; $g = 1/15$; $\sigma = 2$; $q_M = 0$; $p_M = 0.5$; $\alpha = 0.0075$; $\psi = 0.5$; $c = 0$. Panel (b) illustrates the difference in average time spent reproductively mature between the mutant and the wild type (time spent by mutant minus time spent by wild type) for the different scenarios illustrated in panel (a). For details of how the time

spent in each class is calculated, please see Appendix S1, section 2. Panel (c) illustrates the difference in the time spent virulently infected whilst reproductively mature (class V_2) between the mutant and the wild type (time spent by mutant minus time spent by wild type) for the different scenarios illustrated in panel (a). The colours of the lines in panels (b) and (c) have the same meanings as those in panel (a).
