# Supplementary material for: Adaptive immunity selects against malaria infection blocking mutations
Source: PLoS Comput Biol. 2020 Oct 8;16(10):e1008181. doi: 10.1371/journal.pcbi.1008181 (PMC7544067; doi:10.1371/journal.pcbi.1008181)
Supplement: S5 Fig — We illustrate the effect of varying θ on RM under three different infection transmission scenarios, using the supplementary model (see S1 text section 3). The black line is equivalent to the scenario shown in Fig 4A of the main text, where both virulent infections and non-virulent infections transmit at the same rate (βV and βN = 10.20). The blue line shows the case where virulent infections transmit more than non-virulent infections (βV = 30.6 and βN = 10.20). The red line illustrates a scenario where non-virulent infections transmit more than virulent infections (βV = 10.20 and βN = 30.6). Other parameters were as follows: μ1 = 1/30; μ2 = 1/30; g = 1/15; σ = 2; qM = 0; pM = 0.5; α = 0.0075; ψ = 0.5; c = 0. (PDF) [file pcbi.1008181.s006.pdf]

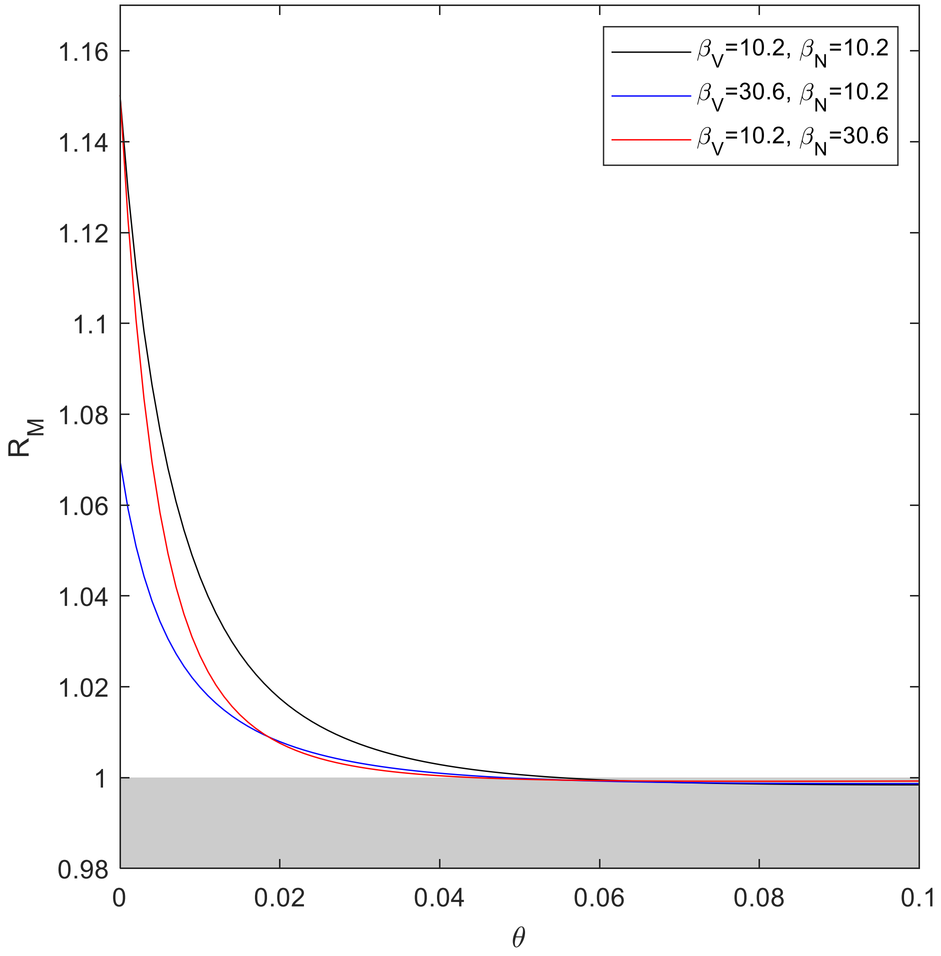

Figure S5: Altering our assumption of uniform transmission from virulence and mild infection does not affect the overall relationship between the rate of gaining virulence immunity (θ) and the success of blocking mutations (R_M). We illustrate the effect of varying θ on R_M under three different infection transmission scenarios, using the supplementary model (see S1 text section 3). The black line is equivalent to the scenario shown in figure 4a of the main text, where both virulent infections and non-virulent infections transmit at the same rate (β_V and β_N = 10.20). The blue line shows the case where virulent infections transmit more than non-virulent infections (β_V = 30.6 and β_N = 10.20). The red line illustrates a scenario where non-virulent infections transmit more than virulent infections (β_V =10.20 and β_N = 30.6). Other parameters were as follows: μ_1= 1/30; μ_2= 1/30; g=1/15; σ=2; q_M=0; p_M=0.5; α=0.0075; ψ=0.5; c=0.
