# Supplementary material for: Adaptive immunity selects against malaria infection blocking mutations
Source: PLoS Comput Biol. 2020 Oct 8;16(10):e1008181. doi: 10.1371/journal.pcbi.1008181 (PMC7544067; doi:10.1371/journal.pcbi.1008181)
Supplement: S6 Fig — Panels (a-i) indicate the time taken for FY*O to reach a frequency ≥90% from a starting frequency of 0.1%, using the extended model (see Methods). We investigate different rates of gaining virulence immunity (θ, x axes), and different properties of FY*O. We assume the FY*O homozygote blocks 59% of infections (phom = 0.59). From left to right across the figure, the infection blocking ability of the FY*O heterozygote increases (phet), and from the top to the bottom row of the figure the protection against virulence afforded by any genotype containing FY*O increases (qhet and qhom). Three different virulence scenarios have been included (see legend). In the low infection costs scenario, α = 0.0001 and ψ = 0.025; in the moderate infection costs scenario, α = 0.0005 and ψ = 0.1, and in the high infection costs scenario, α = 0.0075 and ψ = 0.5. The grey shaded region of each graph indicates unrealistic times (>49000 years). Other parameters were as listed in Table 2, or else were as follows: g = 1/15, r = 0.6, c = 0, β took values between 24.4 and 24.5 so as to keep R0 = 12. (PDF) [file pcbi.1008181.s007.pdf]

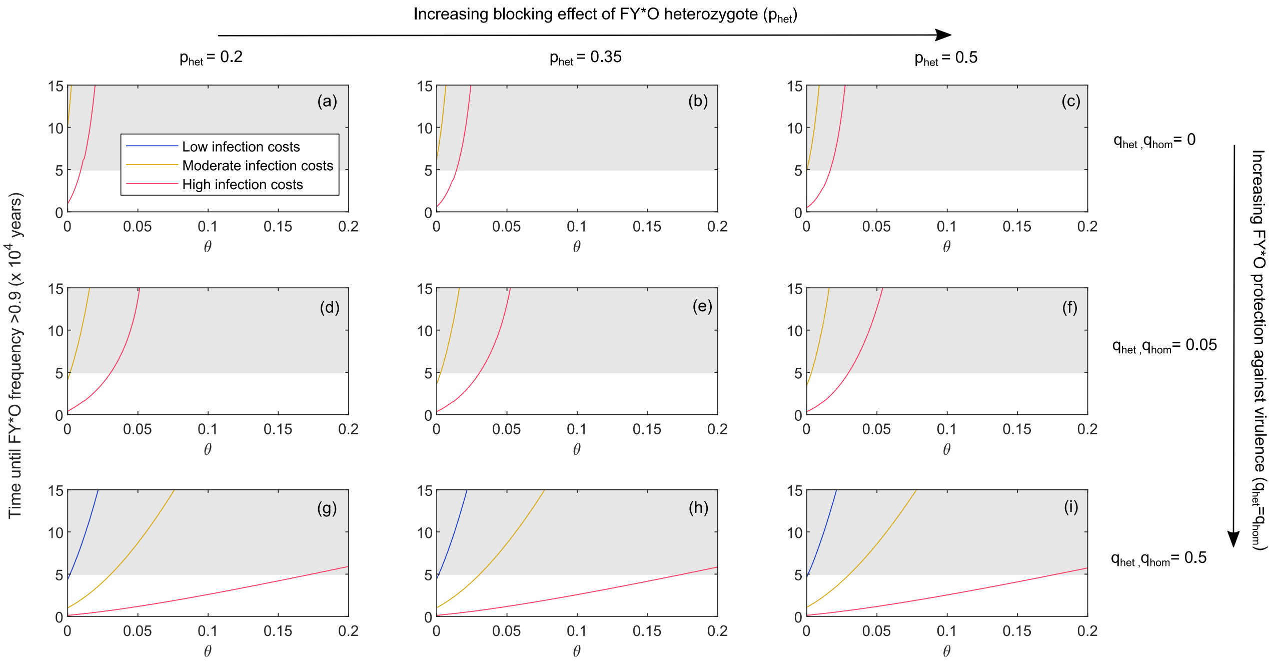

Figure S6: Time taken for FY*O to reach frequencies ≥ 90%, assuming a lower blocking ability of the FY*O homozygote. Panels (a-i) indicate the time taken for FY*O to reach a frequency ≥90% from a starting frequency of 0.1%, using the extended model (see Methods). We investigate different rates of gaining virulence immunity (θ, x axes), and different properties of FY*O. We assume the FY*O homozygote blocks 59% of infections (p_{hom}=0.59). From left to right across the figure, the infection blocking ability of the FY*O heterozygote increases (p_{het}), and from the top to the bottom row of the figure the protection against virulence afforded by any genotype containing FY*O increases (q_{het} and q_{hom}). Three different virulence scenarios have been included (see legend). In the low infection costs scenario, α=0.0001 and ψ=0.025; in the moderate infection costs scenario, α=0.0005 and ψ=0.1, and in the high infection costs scenario, α=0.0075 and ψ=0.5. The grey shaded region of each graph indicates unrealistic times (>49000 years). Other parameters were as listed in Table 2, or else were as follows: g=1/15, r=0.6, c=0, β took values between 24.4 and 24.5 so as to keep R_0=12.
