# Supplementary material for: Adaptive immunity selects against malaria infection blocking mutations
Source: PLoS Comput Biol. 2020 Oct 8;16(10):e1008181. doi: 10.1371/journal.pcbi.1008181 (PMC7544067; doi:10.1371/journal.pcbi.1008181)
Supplement: S7 Fig — Panels (a-c) indicate the time taken for FY*O to reach a frequency ≥90% from a starting frequency of 0.1%, using the extended model (see Methods). We investigate different rates of gaining virulence immunity (θ, x axes), and different virulence protection properties of FY*O. From the top to the bottom row of the figure the protection against virulence afforded by any genotype containing FY*O increases (qhet and qhom). In all panels, the FY*O heterozygote blocks 40% of infections (phet = 0.4), and the FY*O homozygote blocks 96% of infections (phom = 0.96). Results are shown for three different values of R0 for malaria (see legend). The grey shaded region of each graph indicates unrealistic times (>49000 years). Other parameters were as listed in Table 2, or were as follows: α = 0.0075, ψ = 0.5, g = 1/15, r = 0.6, c = 0, β took values between 24.4 and 24.5 so as to generate the necessary values of R0. (PDF) [file pcbi.1008181.s008.pdf]

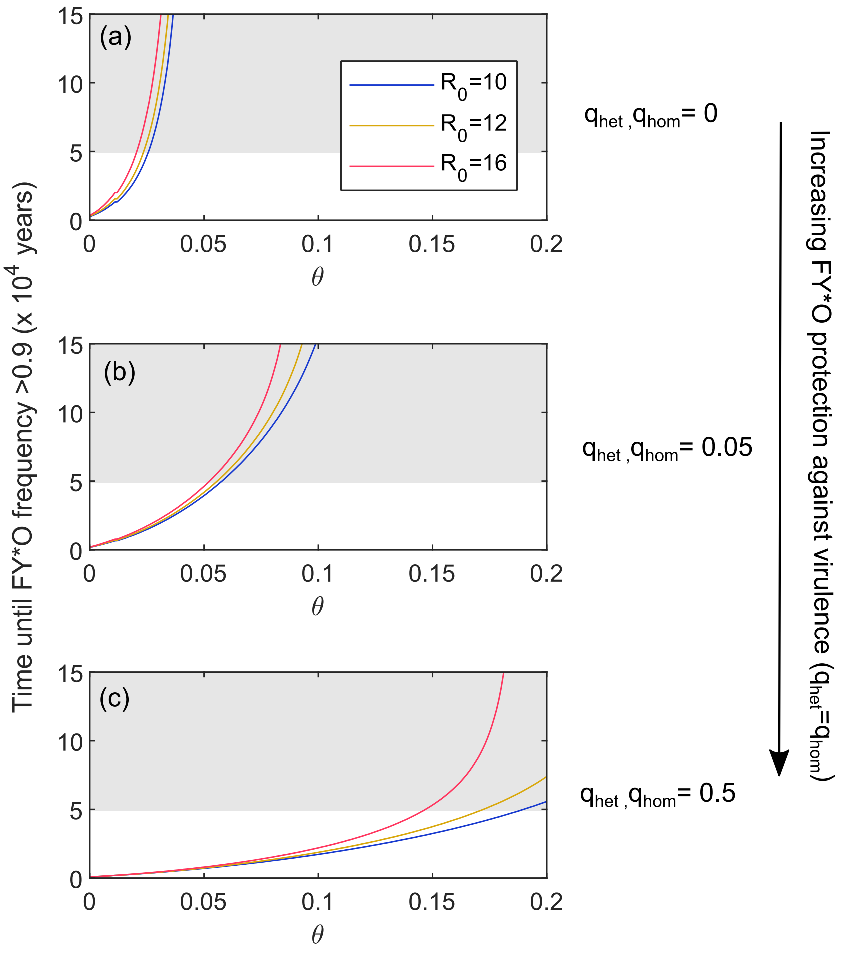

Figure S7: Time taken for FY*O to reach frequencies ≥ 90%, for different values of R_0. Panels (a-c) indicate the time taken for FY*O to reach a frequency ≥90% from a starting frequency of 0.1%, using the extended model (see Methods). We investigate different rates of gaining virulence immunity (θ, x axes), and different virulence protection properties of FY*O. From the top to the bottom row of the figure the protection against virulence afforded by any genotype containing FY*O increases (q_{het} and q_{hom}). In all panels, the FY*O heterozygote blocks 40% of infections (p_{het}=0.4), and the FY*O homozygote blocks 96% of infections (p_{hom}=0.96). Results are shown for three different values of R_0 for malaria (see legend). The grey shaded region of each graph indicates unrealistic times (>49000 years). Other parameters were as listed in Table 2, or were as follows: α=0.0075 , ψ=0.5, *g*=1/15, *r*=0.6, *c*=0, β took values between 24.4 and 24.5 so as to generate the necessary values of R_0.
